# Accurate single-domain scaffolding of three nonoverlapping protein epitopes using deep learning

**DOI:** 10.1038/s41589-025-02083-z

**Published:** 2025-12-05

**Authors:** Karla M. Castro, Joseph L. Watson, Jue Wang, Joshua Southern, Reyhaneh Ayardulabi, Sandrine Georgeon, Stéphane Rosset, David Baker, Bruno E. Correia

**Affiliations:** 1 Institute of Bioengineering, École Polytechnique Fédérale de Lausanne (EPFL), Lausanne, Switzerland; 2 Department of Biochemistry, University of Washington, Seattle, WA USA; 3 Institute for Protein Design, University of Washington, Seattle, WA USA; 4 Imperial College London, London, UK

**Keywords:** Proteins, Protein design, Protein design, Vaccines, Machine learning

## Abstract

De novo protein design has seen major success in scaffolding single functional motifs; however, in nature, most proteins present multiple functional sites. Here, we describe an approach to simultaneously scaffold multiple functional sites in a single-domain protein using deep learning. We designed small single-domain immunogens, under 130 residues, that present three distinct and irregular motifs from respiratory syncytial virus. These motifs together comprise nearly half of the designed proteins; hence, the overall folds are quite unusual with little global similarity to proteins in the Protein Data Bank. Despite this, X-ray crystal structures confirmed the accuracy of presentation of each of the motifs and the multiepitope design yields improved cross-reactive titers and neutralizing response compared to a single-epitope immunogen. The successful presentation of three distinct binding surfaces in a small single-domain protein highlights the power of generative deep learning methods to solve complex protein design problems.

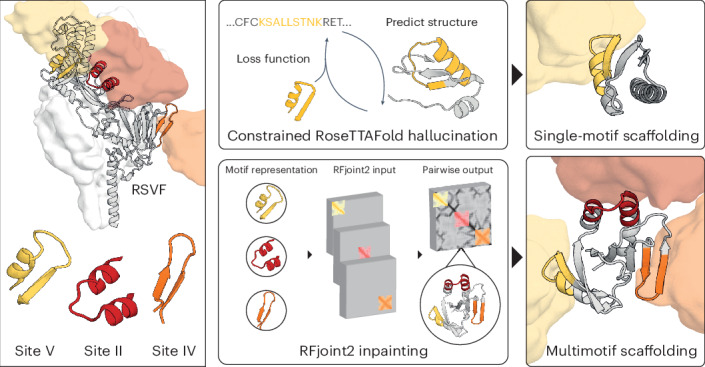

## Abstract (web summary)

A deep-learning-based de novo design strategy was developed that enables simultaneous scaffolding of three distinct epitopes derived from respiratory syncytial virus within small single-domain immunogens. Crystallographic analyses confirmed precise presentation of the designed motifs. The multiepitope constructs elicited enhanced cross-reactive and neutralizing antibody responses, demonstrating the potential of generative models for complex multisite protein engineering.

## Main

Recent advancements in computational protein design have enabled the generation of a huge diversity of protein sequences and structures with atomic accuracy and increasing experimental success^1–4^. Grafting complex structural motifs with traditional computational methods has been restricted to a defined set of characterized protein structures available to host complex motifs, largely limiting our capability to embed designed proteins with function^5^. This has meant that, to date, the majority of de novo proteins are also restricted to a single designed function provided by a single scaffolded motif. In contrast, the majority of natural proteins have multiple functions, often arising as the result of gene fusion^6^. Recent advances in neural networks for both structure prediction and structure or sequence generation enable the design of diverse folds outside of natural protein space^7,8^. Beyond the sampling of complex topologies, deep-learning-powered tools have shown the ability to generate plausible scaffolds embedded with biological functions^9,10^. Deep-learning-based methods largely eliminate the confinement to available protein structure space and have the potential to provide the conformational flexibility required to embed multiple distinct motifs into bespoke de novo scaffolds.

Endowing de novo proteins with multiple functional motifs would enable a much greater diversity of applications. Specifically in vaccinology, the capacity to elicit an immune response to multiple epitopes simultaneously with a single antigen could contribute to the design of better immunogen candidates. Designer proteins displaying viral epitopes promise to improve antibody efficiency by presenting specific neutralizing epitopes to trigger specific immune responses while avoiding non-neutralizing sites with more pronounced immunodominance^11–16^. So-called reverse vaccinology aims to shape the immune response against defined antigenic sites, as viral epitopes are typically irregular and discontinuous structures, making their accurate presentation on de novo proteins a challenging task. Scaffolded immunogenic epitopes have successfully elicited antibodies directed toward the desired sites and, as a cocktail vaccine formulation, can reshape the immune response^14,17,18^. Yet, generating high titers with naive animals using de novo immunogens remains an outstanding challenge. One contributor to this limitation can be attributed to the large fraction of presented surface occupied by the scaffold protein, rather than the valuable immunogenic epitope. Scaffolds presenting multiple nonoverlapping epitopes would largely reduce the nondesired scaffold surface presented in these immunogens.

Here, we use deep-learning-based methods to design de novo scaffolds embedded with up to three functional sites. We create a large, multifaceted antigenic surface composed of three respiratory syncytial virus (RSV) fusion protein (RSVF) epitopes displayed on de novo scaffolds, effectively reducing the nonepitope surface of the scaffold. We demonstrate that all three immunogenic epitopes are recapitulated simultaneously and with a high structural accuracy on de novo generated scaffolds that greatly diverge from previously characterized topological space. Moreover, our multiepitope design shows improved cross-reactive titers to RSVF over a single-epitope immunogen, demonstrating its potential as an alternative avenue for cocktail vaccine formulations. More broadly, we present this approach as a general methodology for the design of multimotif embedded proteins inhabiting largely uncharacterized protein topological space able to host complex motifs applicable to the design of increasingly complex multibinding sites.

## De novo scaffolds presenting a novel RSVF epitope

**Fig. 1:**
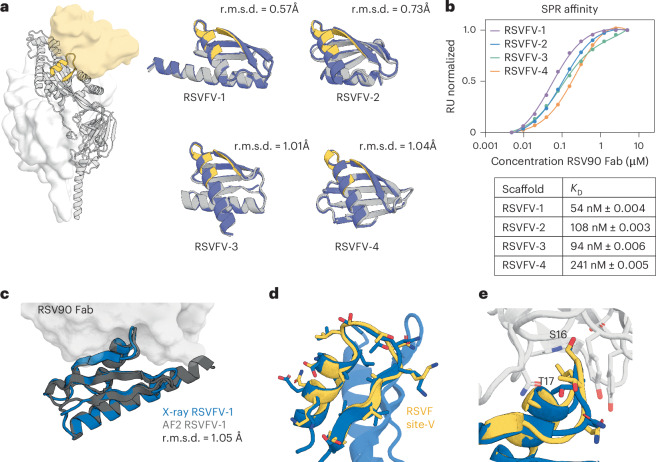
Characterization of RSVFV immunogen candidates. **a**, RSVFV highlighted on the RSVF trimer bound by a site-V-specific antibody (yellow surface). Left, RoseTTAFold hallucination models (gray) of top candidates scaffolding RSVF site V overlaid on AF2 predictions (blue). The all-atom r.m.s.d. is shown for each model. **b**, SPR steady-state affinity measurements for the four designs against RSV90 Fab. The s.e.m. values are reported on the basis of curve fitting. **c**, The crystal structure of RSVFV-1 in complex with RSV90 Fab closely agrees with the AF2 model (r.m.s.d._backbone_ = 1.05 Å). **d**, The RSVFV-1 epitope shows high local similarity to the native RSVF site V structure (r.m.s.d._backbone_ = 0.843 Å). **e**, Superposition of the major contact residues of RSV90 Fab-bound RSVFV-1 superimposed on RSV90-bound RSVF site V (PDB 5TPN; r.m.s.d._all-atom_ = 1.37 Å). Source data

We first explored whether deep learning tools are able to solve single-epitope scaffolding problems that have eluded traditional design methods. The site V epitope of RSVF (RSVFV), consisting of a helix–turn–strand motif, cannot be grafted onto natural scaffolds because of low structural similarity to the Protein Data Bank (PDB) (Supplementary Fig. 1a). In a previous proof of concept, we scaffolded RSVFV into de novo proteins using RoseTTAFold (RF) motif-constrained hallucination, yielding three weak (micromolar) binders to the site-V-specific antibody RSV90 (ref. ^9^). Here, to attempt to obtain higher-affinity designs, we ran a larger-scale hallucination design campaign and additionally incorporated the ProteinMPNN sequence design tool to improve stability and solubility^19^. We screened 4,547 designs and 492 bound to RSV90 at 100 nM using a yeast display binding assay. In total, 27 of 39 chosen hits were expressed in *Escherichia* *coli* and we further characterized four of the best (RSVFV-1 to RSVFV-4), most structurally diverse designs (Fig. 1a). The designs showed good agreement between hallucination models and AlphaFold2 (AF2)^20^ predictions, showed mixed αβ profiles by circular dichroism (CD) spectroscopy, had minimal unfolding up to 90 °C and were mostly monomeric according to size-exclusion chromatography (SEC) coupled to multiangle light scattering (MALS) (Supplementary Fig. 3). The four scaffolds showed *K*_D_ values between 54 and 241 nM to RSV90 (Fig. 1b and Supplementary Table 1), within 50-fold of the RSVF trimer (0.9 nM) (Supplementary Fig. 4) and 20-fold better than the best previous RSVFV design (0.9 μM)^9,21^. We solved the crystal structure of one of the designs, RSVFV-1, in complex with RSV90 Fab to 2.4-Å resolution. The overall structure of the design was in close agreement with its AF2 prediction (root-mean-square deviation (r.m.s.d.)_backbone_ = 1.05 Å) (Fig. 1c) and both the backbone and the side chains of the scaffolded RSVFV epitope closely matched their conformations in the RSV90-bound RSVF structure used for design (motif r.m.s.d.: backbone = 0.843 Å, all-atom = 1.37 Å) (Fig. 1d,e and Supplementary Table 3). Together, these data show that deep learning methods can scaffold antigenic epitopes with high structural accuracy and represent a notable improvement over previous RSVFV single-epitope scaffolding efforts.

## De novo scaffolds incorporating three RSVF neutralizing epitopes

Given the experimental success of deep-learning-based protein design methods on scaffolding epitopes previously considered intractable, we next asked whether multiple epitopes of varying structural complexity could be presented simultaneously on a single scaffold, with the aim of generating immunogens with a higher epitope:scaffold surface area and broader antigenic responses. While computational design approaches have successfully incorporated motifs on de novo scaffolds^5,9,10^, few efforts have been aimed toward engineering single-domain scaffolds presenting multiple distinct epitopes in non-native orientations^5^.

**Fig. 2:**
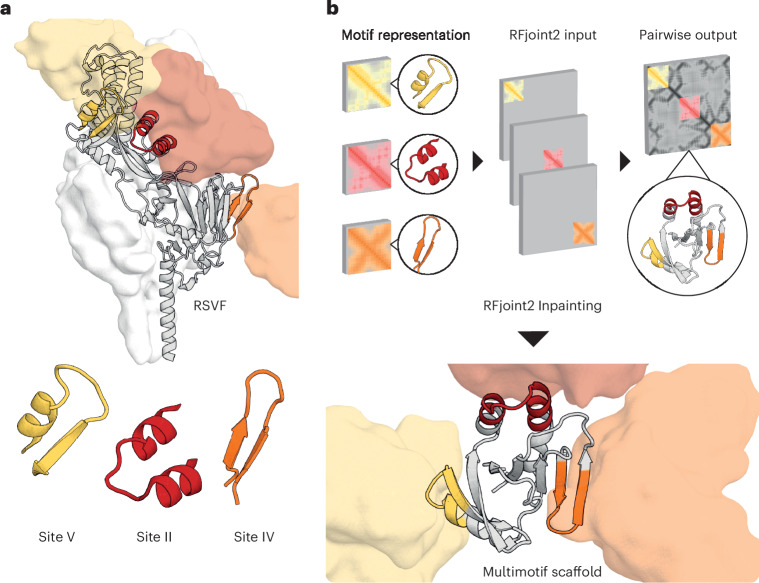
Computational scaffolding of single and multiepitope immunogens. **a**, Epitopes targeted for single-epitope and multiepitope immunogen design highlighted on RSVF structure (PDB 5TPN) and the antibody variable domain against site V (PDB 5TPN), site IV (PDB 3O45) and site II (PDB 3IXT) are represented by the corresponding colored surfaces. **b**, Multimotif scaffolding uses motif templates as input for RFjoint2. Template inputs are invariant to global three-dimensional position; hence, by inputting each motif in a separate template, the relative position of the three motifs is not specified a priori; instead, RFjoint2 builds a scaffold (gray protein) that supports the three motifs (colors) in a non-native position with respect to each other.

**Fig. 3:**
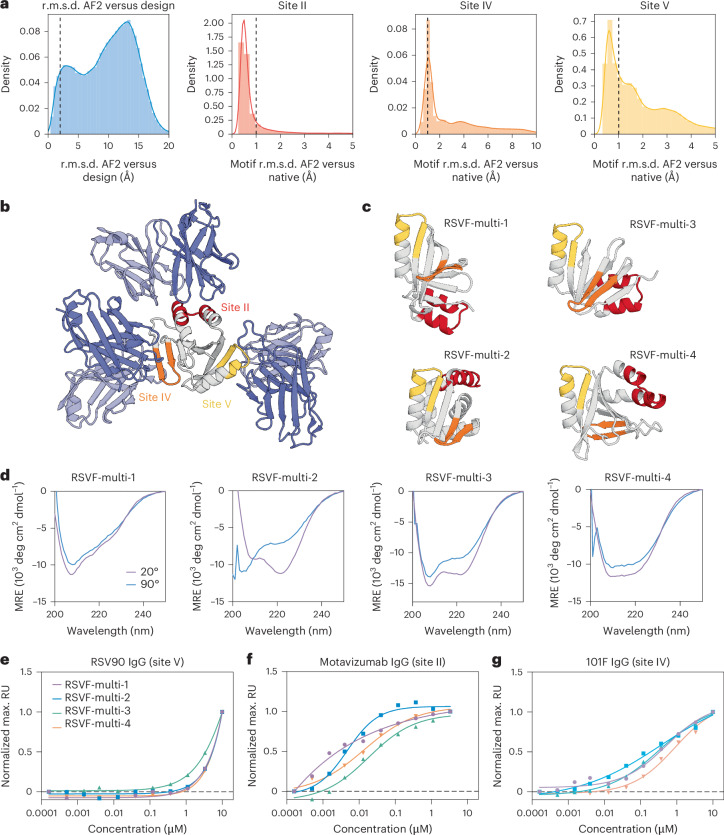
Experimental characterization of multiepitope immunogen candidates. **a**, In silico evaluation. AF2 is used to assess the similarity of predicted structures to the design model and the similarity of each epitope within the prediction to that of the native epitope. Vertical lines: threshold for in silico ‘success’. **b**, Representative design generated with RFjoint2 showing the epitopes remaining accessible to their target antibodies despite this not being explicitly specified during design. The predicted structure of RSVF-multi-4 is aligned to the three known epitope-targeting antibodies (site II, PDB 3IXT; site IV, PDB 3O41; site V, 5TPN). **c**, Predicted structures of top candidate multimotif scaffolds. **d**, CD spectra at start and end incubation temperatures shown for each scaffold. **e**–**g**, Normalized SPR steady-state affinity measurements for four scaffolds for each epitope-specific antibody: RSV90 (site V), motavizumab (site II) and 101F (site IV). Source data

To design scaffolds embedded with three distinct RSVF epitopes known to elicit neutralizing antibodies (Fig. 2a), we used the deep-learning-based RFjoint2 Inpainting method, derived from the RF structure prediction network. We provided fragment templates for sites II, IV and V from RSVF^21–23^, without specifying their relative orientations in the network (Fig. 2b). As these motifs are distinct and, to a large extent, self-contained, their relative orientations are not important for eliciting antibody responses. This flexibility in terms of the rigid-body orientation of the motifs enables the generation of a much greater diversity of scaffolds (Supplementary Fig. 5). Just as for the individual epitope scaffolding with RF, after the initial RFjoint2 Inpainting design step, we used ProteinMPNN^19^ to design the sequence of the scaffold and filtered using AF2 as described previously^10^ (Fig. 3a). To eliminate designs in which the antibodies could not be simultaneously bound to the same scaffold because of steric clashes, we aligned antibodies to the epitopes on the designed scaffold and filtered out those that were structurally incompatible with the antibody-binding modes (Fig. 3b). Because the rigid-body position of the epitopes was not prespecified, RFjoint2 was able to sample a broad range of different epitope positions (Supplementary Fig. 5).

From the filtered multiepitope designs (RSVF-multi), we selected 32 sequences to screen by yeast surface display. To identify folded scaffolds that successfully recapitulated all three epitopes, we initially subjected the library to limited proteolysis and selection with a site-II-specific antibody (motavizumab)^23,24^. The binding population was then subjected to a second round of selection with a site-IV-specific antibody (101F), before a final round of selection with a site-V-specific antibody (RSV90) (Supplementary Fig. 6)^21,22^. From this three-step selection of 32 sequences, we selected five high-frequency recurring sequences. Four of the five designs expressed well in bacteria. CD spectroscopy of four multiepitope designs (RSVF-multi-1, RSVF-multi-2, RSVF-multi-3 and RSVF-multi-4) showed mixed αβ profiles in line with the design models and showed high thermal stability up to 90 °C and mostly monomeric elution profiles (Fig. 3c and Supplementary Fig. 7). All four scaffolds bound to each of the three site-specific antibodies. Quantification of binding affinity for each of the epitopes indicated that both site-IV-specific and site-II-specific antibodies bound all four designs with nanomolar affinities (14–47 nM against a site-II-specific antibody and 343–890 nM against a site-IV-specific antibody) compared to the native RSVF trimer (15 pM to motavizumab Fab and 2 nM to 101F fab, comparable to literature values^21–23^), which may be because of the design challenge of combining three epitopes in one scaffold (Fig. 3e–g, Supplementary Fig. 4 and Supplementary Table 1). The low affinity of the designs for the site-V-specific antibody (>1 μM), compared to the RSVF trimer affinity (0.9 nM) (Supplementary Fig. 4), likely reflects the structural complexity of this epitope (Fig. 3e). However, overall, these results demonstrate for the first time that designs displaying multiple multifunctional sites can be generated with relatively high experimental success rates.

## Multiepitope scaffolds elicit site-specific neutralizing antibodies in vivo

Following successful validation of our multiepitope designs in vitro, we investigated whether, as multiepitope immunogens, they could elicit site-specific responses. We hypothesized that greater immunogenicity might result from immunogens simultaneously displaying multiple different viral epitopes. Previous efforts to recapitulate the RSVF antigenic surface used cocktail vaccine formulations where three neutralizing sites, site 0, site II and site IV, were scaffolded and presented on three different protein structures^14^. For these structures, the relevant epitope residues represent 20–40% of the total surface area of each immunogen, likely resulting in a substantial number of antibodies elicited against the host scaffold. By contrast, the multiepitope designs, by representing the three epitopes on a single scaffold, now represent more than 50% of the total surface area of the immunogen, potentially enabling a superior immune response, especially from the perspective of reducing the decoy surface area presented by the scaffold proteins

**Fig. 4:**
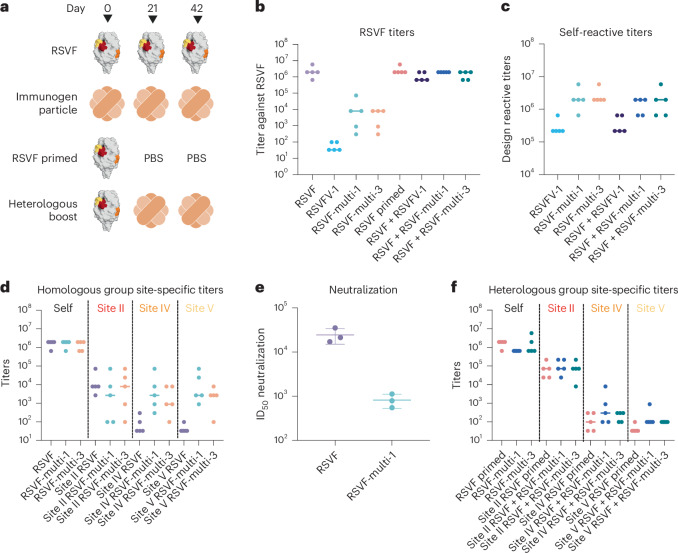
Immunogenicity of single-epitope and multiepitope immunogens. **a**, Four groups of mice (*n* = 5) were immunized with either RSVF or nanoparticle immunogens or a heterologous RSVF primed scheme. **b**, RSVF reactive endpoint titers on day 56 measured by ELISA. Bars indicate the median. **c**, Self-reactive titers of mouse groups immunized with nanoparticle immunogens on day 56. **d**, Site-specific endpoint titers for homologous immunization groups. **e**, RSV serum-neutralizing titers from pooled group sera on day 56. The plot indicates calculated neutralization titers from triplicate experiments after luminescence normalization, with bars indicating the mean and s.d. **f**, Site-specific endpoint titers for heterologous boosted immunization groups. Source data

For in vivo assaying of immune response to the designs, we immunized naive mice with three scaffolds: the single-epitope RSVFV-1 and the multiepitope designs RSVF-multi-1 and RSVF-multi-3. Each scaffold design was incorporated into self-assembling ferritin nanoparticles to improve immunogenicity. Five mice per group were immunized in a homologous boost scheme of three injections of RSVF prefusion trimer or de novo nanoparticle immunogens (Fig. 4a). We alternatively immunized mice with a heterologous prime scheme using RSVF and two boost immunizations with either PBS or nanoparticle immunogens (Fig. 4a). Mice that received RSVF elicited antibody titers and neutralization levels comparable to those of previous studies (Figs. 4b,f)^14,18^. Moreover, consistent with previous studies with single-epitope scaffolded immunogens, the RSVFV-1 immunogen elicited self-reactive titers but low levels of prefusion RSVF cross-reactive titers and no detectable neutralizing activity, indicating that a large proportion of elicited antibodies target the de novo scaffold (Fig. 4b,c)^13,14,18^. However, for both of the RSVF-multi scaffolds tested, we observed moderate levels of prefusion RSVF titers, improved from our single-epitope immunogen by several orders of magnitude (Fig. 4c). Moreover, using single-immunogen designs to probe for epitope-specific reactivity^14^, we observed measurable site-specific titers for both multiepitope immunogen nanoparticle immunizations (Fig. 4d). While the titers of site II were not improved over the RSVF WT immunizations, the titers for site IV and site V were greatly improved. While antibodies targeting the scaffold surface or neoepitopes contribute to the limited overall RSVF titers, these results suggest that the multiepitope scaffolds are able to elicit antibodies with specificity toward all three scaffolded epitopes. Moreover, homologous immunized RSVF-multi-1 sera showed neutralization activity, albeit still less than prefusion RSVF, consistent with previous cocktail vaccine efforts^14^ (Fig. 4e).

Mice receiving multiepitope boosted immunizations showed equivalent titers to RSVF compared to nonboosted mice (Fig. 4b). Additionally, heterologous boosted mice showed equivalent titers to the scaffolds as homologous immunization schemes (Fig. 4c). Interestingly, the site-specific titers are lower than the homologous immunization scheme (Fig. 4f). These results highlight the difficulties of using heterologous scaffolds in prime boosting experiments, where it can be very difficult to boost specific epitope responses.

Through this body of data that include in vivo experiments, we confirmed that deep learning methods can generate protein-based immunogens with a much larger immunogenic surface area compared to single-epitope designs and improved immunogenicity and induction of a physiologically relevant antibody response. These results also validate the previous assumption that the relative surface area of epitope presented is a key determinant of immunogenicity.

## Multimotif inpainted designs are structurally accurate

**Fig. 5:**
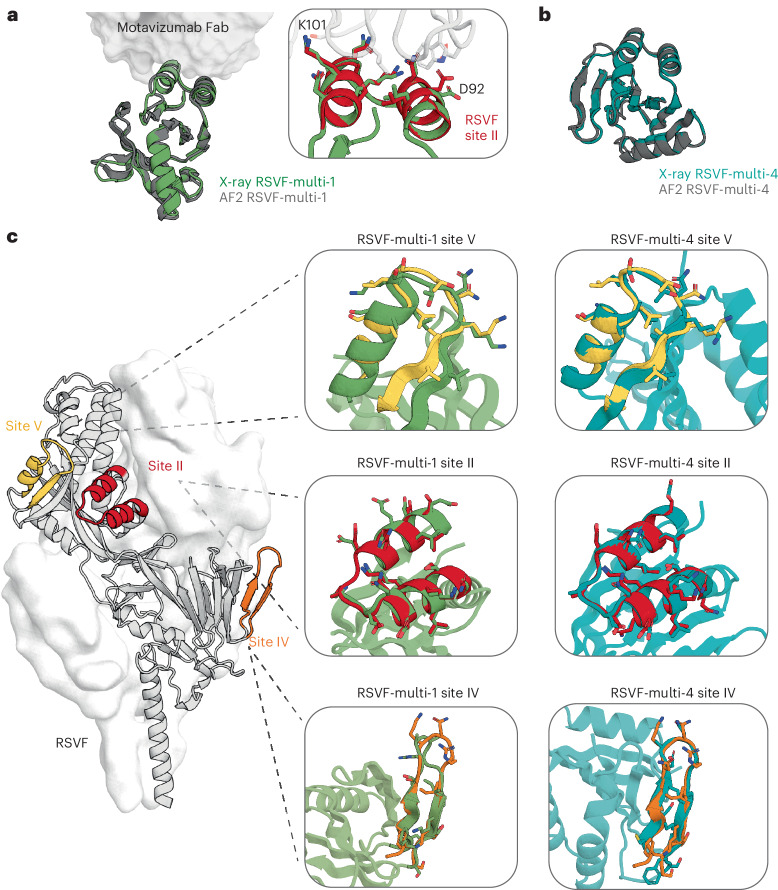
Structural characterization of single-epitope and multiepitope immunogens. **a**, Crystal structure of RSVF-multi-1 (green) bound to motavizumab superimposed on the AF2 model (gray). Inset, close-up view of major contact residues of RSVF-multi-1 superimposed on motavizumab-bound native RSVF site II (red) (PDB 3O45) **b**, Crystal structure of RSVF-multi-4 (teal) overlaid on the AF2 model (gray). **c**, Superimposition of crystal structure epitopes to native site V (yellow), site II (red) or site IV (orange) structures of RSVF (PDB 5TPN).

To evaluate the accuracy of the multimotif designs, we solved the crystal structure of two RSVF-multi immunogens: RSVF-multi-1 in complex with motavizumab Fab and RSVF-multi-4 unbound at 2.91-Å and 2.3-Å resolution, respectively. Both immunogens are in close agreement with the design models indicating that multiepitope design can be achieved with a high degree of structural accuracy (r.m.s.d._backbone_ of approximately 1.4 Å for both designs) (Fig. 5a,b). We first assessed the structural accuracy of the scaffolded epitopes in RSVF-multi-1, where we observed close mimicry of all three RSVF epitopes (Fig. 5c and Supplementary Table 3). While the structure of RSVF-multi-1 shows slight deviation at the C terminus of the target site V backbone structure (r.m.s.d. = 1.776 Å), the major contact residues S29, T30 and N31 (RSVF-multi-1 numbering) and the backbone of K32 are held in the native conformation. The site IV epitope is composed of a flexible β-hairpin and, while the majority of the scaffolded epitope accurately mimics the native site IV epitope, residues 65 and 66 (RSVF-multi-1 numbering) display deviation from the native structure. Lastly, we observe accurate mimicry of the bound site II epitope in the cocrystallized structure with high agreement of side-chain rotamers of conserved scaffolded residues (r.m.s.d._all-atom_ = 1.91 Å) to side-chain rotamers of the antibody-binding interface of native site II and motavizumab Fab complex (PDB 3IXT)^22^ (Fig. 5a).

In the unbound crystal structure of RSVF-multi-4, we observed a high similarity (r.m.s.d._backbone_ < 1.2 Å) of all scaffolded epitopes to the native epitope structures (PDB 5TPN)^21^ (Fig. 5c). Similarly to the structure of RSVF-multi-1, the site IV epitope in one of the well-resolved chains showed good agreement with the native epitope in the context of RSVF (PDB 5TPN) and in the flexible bound epitope peptide (PDB 3O45)^21,22^. The variation in the site IV hairpin structure between the four chains indicated that the flexibility of the epitope can be stabilized by contacts in the crystal lattice (Supplementary Fig. 8). Despite this, there was good alignment of the major antibody contact sites, with an r.m.s.d. of 1.5–2 Å (Supplementary Table 2). As the RSVF-multi scaffolds were not cocrystalized with the site-IV-specific antibody, the greater deviation observed for this epitope may represent an unbound conformation of this loop region outside its native RSVF context. As the structure of RSVF-multi-4 showed close agreement with the native RSVF site V epitope, the low affinity to the site V antibody was unexpected. Five grafted residues not involved in direct contact with the site V antibody were permitted to be redesigned during multiepitope scaffolding. To assess whether these alterations contributed to minor structural integrity of the epitope and its interaction with the site V antibody, rational substitutions were introduced to revert these residues to their native identity and assessed by surface plasmon resonance (SPR) for improved binding to the site V antibody (Supplementary Fig. 9). Indeed, the reverting substitution K29L improved the affinity to the site V antibody (*K*_D_ > 1 μM) and reversion of all five of these residues to native resulted in an improved affinity of *K*_D_ = 0.49 μM. While this magnitude did not reach comparable affinity to RSVF with the site-V-specific antibody^21^, it was comparable to the site-II-specific and site-IV-specific affinity of RSVF-multi-4. This can likely be attributed to the structural complexity of grafting three unique sites on a de novo domain.

Altogether, for all scaffolded epitopes in the X-ray structures obtained, we observed close mimicry, with r.m.s.d. of the backbone and C_β_ to the native epitope below 1 Å in many cases. These results illustrate the capacity of deep learning methods to design accurate de novo scaffolds, even whilst scaffolding multiple distinct functional sites.

**Fig. 6:**
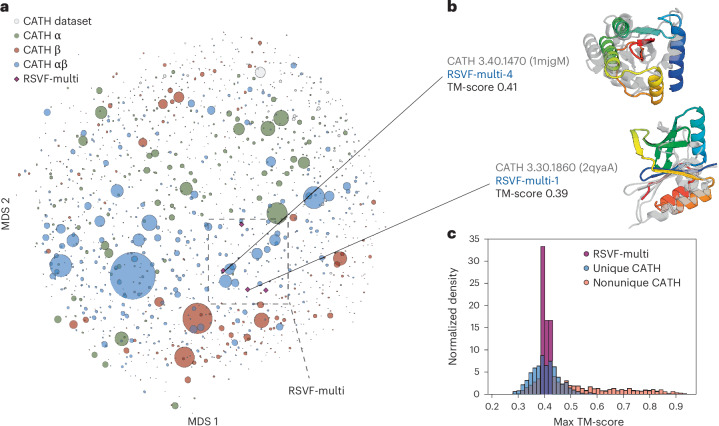
Structural uniqueness of scaffolds presenting single and multiple epitopes. **a**, MDS plot of the pairwise TM-score CATH family representatives and RSVF-multi designs, colored by structure class. Circles represent distinct topologies in CATH, where the relative size is representative of the number of structures in that family. Purple diamonds represent the four multiepitope designs. **b**, Structurally confirmed multiepitope designs (RSVF-multi-1 and RSVF-multi-4) aligned to a representative of the closest CATH topology family. **c**, Maximum TM-score distribution of generated representations of unique versus nonunique topologies against CATH topology families. The RSVF-multi design TM-scores to the closest structural homolog fall within the unique topology distribution. RSVF-multi-1 and RSV-multi-4 are shown aligned to a representative of the closest CATH topology family.

Because of the constraints of scaffolding multiple irregular epitopes, the overall folds of the designs are quite unusual. Comparison to the PDB showed that the closest previously known protein structures had template modeling scores (TM-scores) of 0.46 or lower (0.5 is generally considered to be the threshold for a different fold^25^). The four RSVF-multi designs are also individually distinct from each other (TM-score < 0.52), indicating that incorporation of all three epitopes did not limit the designable topological space. When clustered by pairwise TM-score, all eight designs were distinct from known CATH^26^ topology families (Fig. 6a), with a TM-score to the nearest topology family of 0.39–0.43 (6B). Comparison of the maximum TM-score of our designs to CATH was consistent with the distribution of unique topologies^26^ (Fig. 6c). Taken together, these analyses indicate that the design process created folds novel to the characterized structure space to solve the multimotif scaffolding problem.

## Discussion

The scaffolding of complex motifs has previously been limited by the structural space accessible for design, which has restricted de novo protein design to a one-motif solution per given design problem. In contrast, natural proteins often execute multiple functions with a single chain. Here, we describe the de novo design of scaffolds that host up to three structurally distinct motifs in non-native orientations using deep learning approaches. Deep-learning-based motif scaffolding offers flexibility in the structures and sequences available to accommodate complex epitopes while requiring substantially less user input to find compatible scaffolds, greatly improving accessibility of the de novo motif scaffolding design. In contrast to previous single-motif design involving large libraries and, in some cases, in vitro evolution, we obtained a number of designs binding to all three antibodies using a small number of designed sequences, a notable step toward the generation of multifunctional designs. Improved affinities could likely be achieved through testing alternative topologies and sequences at higher scale. Moreover, we showed that RFjoint2 Inpainting can produce a diverse range of topological solutions for multimotif scaffolding with different relative motif orientations to a high degree of structural accuracy. We were able to achieve accurate local structural similarity to the native epitope structure and functionality of all three epitopes in novel folds dissimilar to structures in the PDB.

As immunogens, presentation of multiple sites simultaneously could offer improved antigenic surface display. The designed multiepitope immunogens elicit higher RSVF cross-reactive titers compared to the site V single-epitope immunogen and improved site-specific reactivity compared to RSVF sera. The heterologous boosted groups did not show improved site-specific titers using single-epitope scaffolds as probes. An alternative approach priming with a multiepitope scaffold and boosting with a different multiepitope scaffold consisting of the same grafted epitopes could electively boost antibodies to the selected epitopes while eliminating antibodies to neoepitopes. Moreover, one of the three-epitope immunogens demonstrated physiologically relevant neutralization titers, implying that the multiepitope immunogens can mediate an immune response against a larger antigenic surface using a single immunogen compared to using a one-epitope immunogen. Previous work demonstrated improvement of RSVF titers by using a cocktail vaccine of single-epitope immunogens^14^. Our multiepitope immunogen can offer an alternative to cocktail vaccines by using a single component, dramatically reducing the cost and improving the speed of production and validation. These multiepitope designs have a much higher proportion of desirable antigenic surface compared to the single-epitope designs, better recapitulating the RSVF antigenic surface on a concise scaffold with reduced potential off-target antibody elicitation.

Beyond immunogens, the ability to incorporate multiple functional sites should be broadly useful for the design of enzymes, sensors and therapeutic candidates. The structural novelty of the designs coupled with the close agreement with the crystal structures is perhaps the most remarkable illustration to date of the ability of generative deep learning methods to build accurate custom solutions to highly constrained designed problems.

## Methods

### Design of site-V-presenting topologies

We designed RSVF site V (PDB 5TPN, residues 163–181) scaffolds using a published RoseTTAFold hallucination method^9^. First, 10,000 designs were hallucinated by 600 steps of gradient descent with a repulsive loss (*α* = 3.5 Å, weight = 2) and a radius of gyration loss (threshold = 16 Å, weight = 1). Half of the designs used a larger definition of the site V motif (chain A residues 163–191) to include an additional β-strand to potentially improve recapitulation of the main motif. Motif residue amino acids were allowed to change except for those in contact with the antibody (residues 165, 166, 169–178, 180 and 188), which were fixed to wild type. A total of 577 designs with AF predicted local distance difference test (pLDDT) > 75, AF motif r.m.s.d. < 1.2 Å and radius of gyration < 16 Å were used as ‘seeds’ for additional iterations of hallucination. Starting from each seed, multiple trajectories of 300–1,000 Markov chain Monte Carlo (MCMC) steps were run with surface nonpolar (weight = 1) and net charge (target charge = −7, weight = 0.02) losses. Designs were filtered on AF pLDDT, AF motif r.m.s.d. and spatial aggregation propensity (SAP) score and used to seed additional rounds of MCMC. Finally, designs were subjected to 20–100 steps of MCMC in the presence of the target antibody to eliminate side-chain clashes with the antibody from residues outside the epitope motif.

A total of 47,498 designs were generated over 14 rounds of hallucination; these were filtered down to 5,717 designs by the following metrics: AF pLDDT > 82, AF motif r.m.s.d. < 1.0 Å, hallucination motif r.m.s.d. < 1.0 Å, AF hallucination whole-protein r.m.s.d. < 2.0 Å, radius of gyration < 16 Å, SAP score < 35, net charge < −5, Rosetta score per residue < −2.5, Rosetta ddG < −10. ‘Motif r.m.s.d.’ refers to the r.m.s.d. over backbone atoms (N, Cα and C) between either the AF prediction or the hallucination (RoseTTAFold-predicted structure on the last step of gradient descent or MCMC) and the input crystal structure motif. Rosetta ddG was calculated after superimposing the design on the native motif in complex with hRSV90 antibody and minimizing side chains in Rosetta. This set of designs was further reduced to 3,072 by clustering at 90% sequence identity using MMseqs2 (ref. ^27^) and keeping one design from each cluster with the lowest mean of AF and hallucination motif r.m.s.d. This comprised the ‘hallucination-only’ subset of the experimentally tested designs.

The ProteinMPNN subset of designs were created by clustering the final hallucinations at 70% sequence identity, to 911 designs, combining with 230 high-scoring seed hallucinations (from gradient descent hallucination before iteration) and inputting their AF2 (ref. ^20^) models to ProteinMPNN^19^ for sequence redesign with temperature = 0.1 and fixing interface residue amino acids. The target antibody was not included in ProteinMPNN design. Eight sequences were generated from each input backbone, yielding 9,128 designs. A total of 592 designs were chosen for testing using the same metric thresholds as above but slightly stricter on motifs (AF motif r.m.s.d. < 0.8 Å, hallucination motif r.m.s.d. < 0.8 Å). Finally, high-scoring ProteinMPNN designs were subjected to Rosetta-based substitution of surface residues to adjust net charge to −7, as this was reported to improve the success rate of designed protein binders^28^; the designs were then filtered using the same thresholds as above and clustered at 90% sequence identity to obtain 883 designs for testing. The yeast display screen showed that ProteinMPNN without adjusting charge was the most successful design method, with a success rate at tenfold binding enrichment of 33.7%, compared to 3.3% for hallucination-only and 21.7% for ProteinMPNN and charge adjustment (Supplementary Fig. 2a).

When selecting designs for experimental testing, we filtered on in silico metrics using thresholds that were chosen by intuition. After experimental testing, we examined the receiver operating characteristic area under the curve (AUC) between each metric and binding success at onefold or tenfold enrichment between binding and nonbinding yeast populations (Supplementary Fig. 2b,c)^29,30^ AUC was relatively high (between 0.7 and 0.8) for several metrics when considering all designs but within each of the three design methods, AUC was much lower and usually close to random (0.5). This suggests that most of the differences in success rate were driven by the design method and more stringent filtering on the metrics we used would not have further increased success.

### Designing multiepitope scaffolds without specifying interepitope rigid-body orientation

We sought to develop a design methodology to permit the scaffolding of multiple epitopes into a single designed protein. Importantly, because each individual epitope can be considered its own unit, we want a methodology whereby the rigid-body orientation between the epitopes can be sampled or chosen during design. In other words, we do not want to keep the native rigid-body orientation nor do we want to have to prespecify this rigid-body orientation.

We, therefore, developed an approach using RFjoint2 Inpainting, taking advantage of its ability to accept multiple independent ‘templates’ (inherited from the original RF model from which it is fine-tuned). During protein structure prediction, RF uses any available homologous structures to aid it in structure prediction. Importantly, there can be multiple templates, which align to distinct and nonoverlapping regions of the query sequence. Hence, these multiple templates are provided to RF in a rigid-body orientation-independent manner, such that the rigid-body orientation between the two templated regions is not specified or fixed during prediction (because it is unknown). This invariance comes from the pairwise representation with which templates are provided to RF. Within each template, the pairwise residue–residue distances and dihedral angles^29^ are provided to the network. The pairwise distances and angles between templates are, therefore, not provided to RF.

We exploited this in RFjoint2 Inpainting to develop a method whereby individual epitopes could be provided as individual templates to the network. In other words, the three RSVF epitopes we scaffolded (sites II, IV and V) were each provided to the network as their own distinct template input. Thus, RFjoint2 Inpainting was able to see each epitope’s internal structure but not the rigid-body orientation between them. RFjoint2, therefore, had to simultaneously build a scaffold (with the specified masked residues that connected the epitopes) and ‘choose’ the rigid-body orientation between the three epitopes. The ability to perform this task is inherent to RFjoint2 Inpainting and did not require further training.

### Designing multiepitope scaffolds scaffolding RSV sites II, IV and V

#### Backbone generation with RFjoint2

The structures of sites II, IV and V were extracted from PDB 4JHW. The three sites were defined as described in Supplementary Table 5

Because the rigid-body orientation between epitopes is not specified, there is no guarantee that designs will leave each epitope accessible to the target antibody. Pilot experiments demonstrated that multiepitope designs often had detrimental clashes with the 101F site IV antibody (from PDB 3O41). Therefore, to prevent this, we additionally included residues 1–116 of the 101F Fab (from PDB 3O41) in the site IV input template.

To generate a diverse set of designs, the order of the three epitopes in the designs was enumerated and the lengths connecting each epitope were systematically sampled (10–20 aa at the N and C termini and connecting the epitopes). A total of 17,475 designs were made (approximately 20% of the total possible enumerated design inputs with these parameters). In all cases, the ‘template confidence’ (that is, how confident RFjoint2 should be in the internal structure of each input epitope) was set to 1.

#### Sequence design with ProteinMPNN

Although RFjoint2 Inpainting simultaneously designs a structure and sequence, the ability of ProteinMPNN to very rapidly design multiple unique sequences for a given protein backbone renders it preferable over the single sequence that RFjoint2 produces for a given protein backbone. Therefore, we used ProteinMPNN for the sequence design step of the design pipeline.

To reduce the compute burden, we prefiltered RFjoint2 outputs before downstream sequence design and filtering. Specifically, we filtered both on the internal confidence metric (pLDDT) within RFjoint2 (>0.6) and on the ‘compactness’ of the outputs (radius of gyration, aspect ratio and the maximum distance between any two residues).

With these filtered backbones, we designed 16 sequences for each backbone with ProteinMPNN^19^, with default parameters. Because not all of the residues within each epitope are actually involved in binding the target antibodies, we allowed ProteinMPNN to redesign the residues described in Supplementary Table 6.

#### Repredicting the structure with AF2

Following numerous other works^9,10,31^, we predicted the structure of the sequences out of ProteinMPNN using AF2 to determine the similarity of the AF2 prediction and the designed protein backbone (the ‘self-consistency’). We, therefore, predicted the structure of the sequences using AF2_model_4_ptm, with three recycles.

#### Calculating interface metrics with Rosetta

In addition to filtering on AF2 self-consistency, we filtered on the quality of the interface with each target antibody, as assessed by Rosetta^32^. For this, we aligned the AF2 predictions of each design onto the original antibody–epitope structure (site II, PDB 3IXT; site IV, PDB 3O41; site V, PDB 5TPN). Following Rosetta FastRelax^32^, we calculated the predicted ddg of the interface.

#### Filtering designs for experimental characterization

For testing, we filtered designs using the following metrics, which largely follow previous work^9,10,28^:

- Epitope r.m.s.d. (AF2 versus native) < 1 Å
- Overall r.m.s.d. (AF2 versus design model) < 2 Å
- AF2 pLDDT > 80
- Rosetta ddg for each epitope aligned to its respective antibody < −10
- Rosetta SAP score < 40

### Yeast library preparation

A DNA oligonucleotide pool for RSVFV designs used constant 3′ and 5′ ends for the amplification and attachment of overhangs for yeast transformation by PCR. The oligo pool was resuspended in H_2_O to 50 ng μl^−1^ and amplified by PCR (55 °C annealing for 30 s, 72 °C extension time for 1 min, 25 cycles) The PCR also allowed for attachment of homologous overhangs for yeast transformation. The PCR product was desalted and transformed together with a linearized pCTcon2 vector as previously described^33^ into the EBY-100 yeast strain with a transformation efficiency of at least 10^6^. The RSVF-multi library genes were cloned into pETcon3 vector and transformed into chemically competent yeast as described by Geitz and Schiestl^34^ scaled to a total transformation volume of 60 μl.

### Yeast surface display

The transformed cells were passaged twice in SDCAA medium with penicillin and streptomycin before induction in SGAA medium overnight at 30 °C. Induced cells were pelleted by centrifugation (3,000*g*, 3 min). Pellets corresponding to 2 ml at an optical density at 600 nm (OD_600_) of 1 were washed once and resuspended 250 μl in TBS (20 mM Tris pH 8.0 and 150 mM NaCl). Chymotrypsin was diluted in 250 μl at 2× final concentration. Final concentrations of chymotrypsin were 0.01 μM (pCTcon2 vector) or 0.1 μM trypsin (pETcon3 vector). Chymotrypsin was added to the resuspended yeast and incubated at room temperature for 5 min. The reaction was quenched in cold PBS + 2% BSA. Cells were pelleted and washed in cold wash buffer (PBS + 0.05% BSA) three times. Cells were labeled with 1 µM of the target (RSV90 Fab for RSVFV library, motavizumab IgG for the RSVF-multi library) at 4 °C for 2 h. Cells were washed twice with wash buffer and then incubated with FITC-conjugated anti-HA (pCTcon2 vector) or FITC-conjugated anti-myc (pETcon3 vector) and PE-conjugated anti-human Fc (BioLegend, 342303) or PE-conjugated anti-Fab (Thermo Scientific, MA1-10377) for an additional 30 min. Cells were washed and sorted using a SONY SH800 flow cytometer in ‘ultrapurity’ mode. Cells were grown in SDCAA medium. The RSVFV library was subjected to a second sort at 1 µM 101F Fab. Following the sorts, cells were plated on SDCAA agar and more than 40 single colonies were sequenced to obtain 19 candidates for biochemical characterization. The RSV multiepitope library was subject to a second sort at 1 µM RSV90 IgG and third sort at 1 µM RSV90 IgG to screen against all three antibodies. Cells from the third sort were plated and 40 single colonies were sequenced, for which the converged five sequences were selected as candidates for biochemical characterization.

### MiSeq sequencing

After sorting, cells were passaged once in SDCAA medium with penicillin and streptomycin. The cells were pelleted and lysed for DNA extraction using Zymoprep yeast plasmid miniprep II (Zymo Research) using the manufacturer’s instructions. The extracted DNA was amplified using plasmid-specific primers and standard Illumina sequencing adaptors were attached with overhang PCR in reaction I. Illumina sequencing primers with unique barcodes were added in PCR reaction II. PCR products from both reactions were cleaned using AMPure XP bead-based reagent (Beckman Coulter). Eluted DNA was validated by fragment analyzer before submission for Illumina MiSeq by the Gene Expression Core Facility (École Polytechnique Fédérale de Lausanne, EPFL). A total of 500 paired-end cycles were run with approximately 2 million reads per sample. Sequences were trimmed and translated in the correct reading frame for bioinformatic analysis and fold enrichment was computed for each sequence as the fraction counts in the binding population over the nonbinding population. Binding designs were considered if a tenfold enrichment in binding versus nonbinding populations was observed. Designs for biochemical characterization were selected among reoccurring sequences from MiSeq in combination with single-colony sequencing. Designs whose models were structurally diverse were selected.

### SPR

SPR measurements were performed on a Biacore 8K (GE Healthcare) in 10 mM HEPES pH 7.4, 150 mM NaCl, 3 mM EDTA and 0.005% v/v surfactant P20 (GE Healthcare). Scaffolds and RSVF trimer or IgG proteins were immobilized on a CM5 chip (GE Healthcare, 29104988) by amine coupling. Approximately 1,000 response units of protein were immobilized and antibody Fab or designed monomeric proteins were injected as analyte in threefold or sixfold serial dilutions. The flow rate was 30 μl min^−1^ for a contact time of 120 s followed by a 600-s dissociation time. After each injection, the surface was regenerated using 0.1 M glycine at pH 3.0. Kinetic data were fitted using a 1:1 Langmuir binding model within the Biacore 8K analysis software (GE Healthcare, 29310604). Steady-state affinity was normalized and fitted using a GraphPad one-site binding model. The s.e.m. was calculated on the basis of curve fitting.

### CD

CD spectra were measured using a Chirascan instrument in a 1-mm-path-length cuvette. The protein samples were prepared in 10 mM sodium phosphate buffer at a protein concentration of ~30 μM. Wavelengths between 195 nm and 250 nm were recorded with a scanning speed of 20 nm min^−1^ and a response time of 0.125 s. All spectra were corrected for buffer absorption. Temperature ramping melts were performed from 20 to 90 °C with an increment of 2 °C min^−1^ or 5 °C min^−1^. Thermal denaturation curves were determined by the change in ellipticity minimum at 220 nm.

### MALS

Samples containing of protein between 0.7 mg ml^−1^ and 1 mg ml^−1^ in PBS buffer (pH 7.4) were injected into a Superdex 75 10/300 GL column (GE Healthcare) at a flow rate of 0.5 ml min^−1^ coupled inline to an MALS device (miniDAWN TREO, Wyatt). Scatter data were analyzed by ASTRA software (Wyatt).

### Immunogen expression and purification

Genes encoding scaffolds were purchased as DNA fragments from Twist Bioscience and were cloned into pET11b bacterial expression vector. Plasmids were transformed into *E*. *coli* BL21 (DE3). Following overnight preculture growth, cultures were grown in Terrific Broth autoinduction medium at 37 °C to an OD_600_ of 0.7 and then transferred to 18 °C for 16 h. Cells were harvested and pellets were resuspended in lysis buffer (50 mM Tris pH 7.5, 500 mM NaCl, 5% glycerol, 1 mg ml^−1^ lysozyme, 1 mM PMSF and 1 μg ml^−1^ DNase) supplemented with 1× CellLytic B cell lysis reagent (Merk). Chemical lysis was performed rotating at 4 °C for 1 h. Lysates were clarified by centrifugation (48,000*g*, 20 min) and purified by Ni-NTA affinity chromatography eluting with 10 mM Tris, 500 mM NaCl and 300 mM imidazole (pH 7.5) and subsequent SEC on a HiLoad 16/600 Superdex 75 column (GE Healthcare) in PBS buffer.

### RSVF protein expression and purification

The prefusion RSVF sc9-10 DS-Cav1-A149C;Y458C;S46G;E92D;S215P;K465Q thermostabilized variant was previously cloned and annotated as DS2 (refs. ^18,35^). The plasmid was transfected in ExpiCHO cells. Supernatants were harvested after 7 days and purified by Ni-NTA affinity chromatography eluting using 10 mM Tris, 500 mM NaCl and 300 mM imidazole (pH 7.5). The eluted protein was further purified on a StrepTrap HP affinity column (GE Healthcare) and eluted using 10 mM Tris, 150 mM NaCl and 20 mM desthiobiotin (pH 8) (Sigma). Final purification was performed using SEC in PBS (pH 7.4) on a Superdex200 Increase 10/300 GL column (GE Healthcare).

### Nanoparticle-linked immunogen expression and purification

The target scaffold genes for RSVFV-1, RSVF-multi-1 and RSVF-multi-3 were fused upstream of a gene encoding *Helicobacter* *pylori* ferritin (GenBank ID: QAB33511.1) and N-terminal 6×His tag. The fusion consisted of a GGSGGSGG, GNGSGGNGSGAEAAAKEAAAKAGNGSGGNGS or GTGGSGGSGG linker. The fused genes were cloned into the pHLsec vector for mammalian expression. Ferritin-linked immunogens were transfected in HEK-293T cells. The supernatant was harvested after 6 days and purified using Ni-NTA affinity chromatography and SEC on a Superose 6 increase 10/300 GL column (GE).

### Antibody IgG and Fab protein expression and purification

Heavy-chain and light-chain DNA sequences of Fab were purchased from Twist Bioscience and cloned individually into the pHLsec mammalian expression vector (Addgene, 99845) using Gibson assembly. The heavy-chain sequence was additionally cloned into pHLsec-Fc containing the human C_H_2 and C_H_3 region. HEK-293T cells were transfected with a 1:3 ratio of heavy to light chain. Supernatants were collected after 6 days and purified using a 5-ml HiTrap Protein A HP column (GE Healthcare) for IgG expression and 5-ml kappa-select column (GE Healthcare) for Fab purification. IgG or Fabs were eluted with 0.1 M glycine buffer (pH 2.7), immediately neutralized by 1 M Tris buffer (pH 9) and further purified by SEC on a Superdex200 Increase 10/300 GL column (GE Healthcare) in PBS.

### Mouse immunizations

All animal experiments were approved by the Vaud Veterinary Cantonal Authorities in accordance with Swiss regulations of animal welfare (VD3808). Female BALB/c mice at 5 weeks old were obtained from Janvier Labs and acclimatized for 1 week. Mice were housed at 22 ± 2 °C and 55% ± 10% relative humidity. The mice were under a 12-day 12-h night cycle, starting at 7:00 a.m. and ending at 7:00 p.m. in four-stage intensity changes. Immunogens were mixed with equal volumes of adjuvant (Alhydrogel, Invivogen) and incubated for 1 h on ice. Mice were injected subcutaneously with 100 μl of vaccine formulated with 5 μg of immunogen. Immunizations were performed on days 0, 21 and 42. Blood samples were collected on days 0, 14 and 35. Mice were killed on day 56 and blood collection was performed by cardiac puncture.

### ELISA

Purified recombinant RSVF trimer or monomeric epitope immunogens were coated on 96-well plates (Nunc MediSorp, Thermo Scientific) overnight at 4 °C with 0.5 μg ml^−1^ in coating buffer (PBS pH 7.4). Plates were washed three times at each step with wash buffer (PBS + 0.05% Tween-20 (PBS-T)) Wells were blocked with blocking buffer (PBS-T with 5% skim milk (Sigma)) for 1 h at room temperature. Threefold serial dilutions were prepared in assay buffer (PBS-T + 1% BSA) and were added to the plates and incubated at room temperature for 2 h. Secondary incubation with anti-mouse (Abcam, 99617) horseradish-peroxidase-conjugated secondary antibody diluted 1:1,500 was added and incubated for 1 h at room temperature. Plates were developed by adding 100 μl of TMB substrate (Thermo Scientific). The reaction was stopped using an equal volume of 0.5 M HCl. The absorbance at 450 nm was measured on a Tecan Safire 2 plate reader. The endpoint titer was determined as the reciprocal of the serum dilution that resulted in a signal twofold above background.

### Neutralization assay

RSV neutralization was performed as previously described^8^. Hep2G cells were seeded in Corning 96-well tissue culture plates (Sigma) at 40,000 cells per well overnight at 37 °C at 5% CO_2_. Serial dilutions of heat-inactivated sera were prepared in M0 medium (EMEM without phenol red, supplemented with 2 mM L-glutamine, 100 IU per ml penicillin and 100 µg ml^−1^ streptomycin) and incubated with 100 plaque-forming units per well of RSV-Luc (RSV long strain carrying a luciferase gene) for 1 h at 37 °C. The serum with virus was added to the cell monolayer and incubated for 48 h at 37 °C. Cells were lysed in 100 μl of lysis buffer (31.25 mM Tris pH 7.9, 10 mM MgCl_2_, 1.25% Triton X-100 and 18.75% glycerol) for 10 min at room temperature. Then, 50 μl of lysate was transferred to a white 96-well plate (Grenier). An equal volume of assay buffer (lysis buffer, supplemented with 1 mM DTT, 1.1 μg ml^−1^ luciferin (Sigma, L-6882) and 2 mM ATP (Sigma, A3377)) was added and luminescence was immediately measured on a Tecan Spark plate reader. Plots were normalized for luminescence and fitted using the GraphPad variable slope fitting model, weighted by 1/*Y*^2^.

### X-ray crystallization and structural determination cocrystallization of complex RSV90 Fab with RSVFV-1

#### Cocrystallization

The RSVFV-1 and RSV90 Fab were purified by SEC using a Superdex200 26 600 (GE Healthcare) equilibrated in 10 mM HEPES pH 7.5 and 150 mM NaCl and concentrated to ~20 mg ml^−1^ (Amicon Ultra-15, molecular weight cutoff (MWCO) = 3,000). The immunogen and Fab were mixed in equimolar quantities and incubated at 4 °C for 1 h. Crystals were grown at 18 °C using the sitting-drop vapor-diffusion method in drops containing 200 nl of protein complex and 200 nl of reservoir solution containing 0.1 M HEPES pH 7.5 and 30% (v/v) PEG Smear Low. Crystals appearing after 5 days were crushed and used for seeding. Crystals were grown at 18 °C in drops containing 200 nl of protein complex, 200 nl of reservoir solution and 50 nl of seeding crystal solution. Crystals appeared in condition solution containing 0.1 M MES pH 6.5 and 20 % (v/v) PEG Smear High. For cryoprotection, crystals were briefly immersed in mother liquor containing 20% ethylene glycol.

#### Data collection and structural determination

Diffraction data were recorded with X06SA (PXI) at the Swiss Light Source. The diffraction data were integrated and processed to 2.43 Å by AutoProc^36^ with a high-resolution cutoff at *I*/*σ* = 1 applied. The crystals belonged to space group *P* 21 21 2. The structure was determined by molecular replacement using the Phaser module in the PHENIX program^37^. The searching of the initial phase was performed using the RSV90 structure (PDB 5TPN) and the AF2 model of RSVFV-1 as a search model. One Fab copy contained loops opposite the interaction interface that could not be built. Manual model building was performed using Coot^38^ and automated refinement was performed using phenix.refine. The final refinement statistics are summarized in Supplementary Table 2.

### X-ray crystallization and structural determination cocrystallization of complex motavizumab Fab with RSVF-multi-1

#### Cocrystallization

The RSVF RSVF-multi-1 immunogen and motavizumab Fab were purified by SEC using a Superdex200 26 600 (GE Healthcare) equilibrated in 10 mM HEPES pH 7.5 and 150 mM NaCl and concentrated to ~20 mg ml^−1^ (Amicon Ultra-15, MWCO = 3,000). The immunogen and Fab were mixed in equimolar quantities and incubated at 4 °C for 1 h. Crystals were grown at 18 °C using the sitting-drop vapor-diffusion method in drops containing 200 nl of protein complex and 200 nl of reservoir solution containing 0.2 M potassium thiocyanate, 0.1 M sodium cacodylate pH 6.5 and 10 % (v/v) PEG 1000.

#### Data collection and structural determination

Diffraction data were recorded with ID30B at the European Synchrotron Radiation Facility. The diffraction data were integrated and processed to 2.692 Å by AutoProc^36^ with a high-resolution cutoff at *I*/*σ* = 1 applied. The crystals belonged to space group *C* 121. The structure was determined by molecular replacement using the Phaser module in the PHENIX program^37^. The searching of the initial phase was performed using the motavizumab structure (PDB 3IXT) and the partial AF2 model of RSVF-multi-1 as a search model. Manual model building was performed using Coot^38^ and automated refinement was performed using phenix.refine. The final refinement statistics are summarized in Supplementary Table 2.

### X-ray crystallization and structural determination of RSVF-multi-4

#### Crystallization

The RSVF-multi-4 immunogen was purified by SEC using a Superdex200 26 600 (GE Healthcare) equilibrated in 10 mM HEPES pH 7.5 and 150 mM NaCl and concentrated to ~20 mg ml^−1^ (Amicon Ultra-15, MWCO = 3,000). Crystals were grown at 18 °C using the sitting-drop vapor-diffusion method in drops containing 200 nl of protein complex and 200 nl of reservoir solution containing 0.2 M calcium acetate, Tris pH 7.5 and 25 % (v/v) PEG 2000 MME.

#### Data collection and structural determination

Diffraction data were recorded with ID30A1 at the European Synchrotron Radiation Facility. The diffraction data were integrated and processed to 2.382 Å by AutoProc^36^ with a high-resolution cutoff at *I*/*σ* = 1 applied. The crystals belonged to space group *P* 21 21 21. The structure was determined by molecular replacement using the Phaser module in the PHENIX program^37^. The searching of the initial phase was performed using the AF2 model of RSVF-multi-4 as a search model. Manual model building was performed using Coot^38^ and automated refinement was performed using phenix.refine. Unresolved loops were not built. The final refinement statistics are summarized in Supplementary Table 2.

### Structure and sequence space analysis

#### Yeast display structural and sequence space analysis

For analyzing the structure space of proteins from the RSVFV yeast library, we calculated an all-versus-all TM-score^25^. This allowed us to assess the structural similarity of the designs belonging to 444 backbone families and to visualize this structural space with multidimensional scaling (MDS). Additionally, we calculated the success rate of each backbone family by calculating the fraction of sequences within each family that were identified in the binding population. The five most successful families were defined as the backbone families consisting of the largest fraction of successful sequences. All sequences within each of the five most successful families were scored for sequence identity to each other using MMseqs2 (ref. ^27^).

#### CATH structural space analysis

To assess the novelty of the eight characterized proteins and their relationship to known topologies, we compared their structural similarity to the CATH database^26^. The CATH dataset partitions proteins into a structural hierarchy: class, architecture, topology and homologous superfamily. To visualize the topological similarity space we used MDS to embed into two dimensions the matrix of all pairwise TM-scores^25^ of the union of our eight immunogens and CATH representatives from each of the 1,472 known topologies. To further assess the novelty, we created two reference distributions of TM-scores against CATH: one for proteins being sampled with a novel topology and another for proteins sampled with a topology that commonly occurs in the dataset. The novel distribution was achieved by removing a topology from CATH and calculating the maximum TM-score between any protein in the excluded topology to all proteins from other topology families. The nonunique distribution was achieved by repeating this process without removing the topology. The third dataset was created by comparing our eight designs to CATH. We compared the maximum TM-scores from our characterized proteins to these reference distributions to identify structurally similar topology families as a proxy for novelty, as previously described^39^.

### Reporting summary

Further information on research design is available in the Nature Portfolio Reporting Summary linked to this article.

## Online content

Any methods, additional references, Nature Portfolio reporting summaries, source data, extended data, supplementary information, acknowledgements, peer review information; details of author contributions and competing interests; and statements of data and code availability are available at 10.1038/s41589-025-02083-z.

## Supplementary information

Supplementary Information Supplementary Figs. 1–9 and Tables 1–6.

Reporting Summary

Supplementary Data 1 Source data for Supplementary Fig. 9.

## Source data

Source Data Fig. 1 Normalized SPR source data.

Source Data Fig. 3 Normalized SPR source data.

Source Data Fig. 4 ELISA data and neutralization plot.

## Data Availability

Structures were deposited to the PDB under accession codes 9F91 (RSVFV-1 in complex with RSV90 Fab), 9F90 (RSVF-multi-1 in complex with motavizumab Fab) and 9F8Y (RSVF-multi-4). Protein sequences are available in Supplementary Table 4. The plasmids of the designed proteins are available from the authors under a material transfer agreement with EPFL. Source data are provided with this paper.

## Code availability

The code for RF2joint is available from GitHub (https://github.com/RosettaCommons/RFjoint2.git).
