# Supplementary material for: Accurate single-domain scaffolding of three nonoverlapping protein epitopes using deep learning
Source: Nat Chem Biol. 2025 Dec 5;22(4):604–11. doi: 10.1038/s41589-025-02083-z (PMC13038408; doi:10.1038/s41589-025-02083-z)
Supplement: Supplementary file 1 — Supplementary Figs. 1–9 and Tables 1–6. [file 41589_2025_2083_MOESM1_ESM.pdf]

nature chemical biology

Supplementary information

https://doi.org/10.1038/s41589-025-02083-z

Accurate single-domain scaffolding of three nonoverlapping protein epitopes using deep learning

In the format provided by the authors and unedited

Supplementary Information

Supplementary Figure 1: Available natural scaffolds to host RSVF epitopes 2
Supplementary Figure 2. Pooled analysis of RSVF site V designed binders 3
Supplementary Figure 3: RSVFV biochemical characterization 4
Supplementary Figure 5: RFjoint2 generates diverse inter-epitope positioning 6
Supplementary Figure 6: Sequential sorting for the identification of multi-epitope scaffolds binding all target antibodies. 7
Supplementary Figure 7: RSVF-multi biochemical characterization 8
Supplementary Table 1: Affinity RSVFV and RSVF-multi designs site-specific antibodies 9
Supplementary Table 2: Structural accuracy of grafted RSVF epitopes on single and multi-motif scaffolds. 9
Supplementary Figure 8: Site-IV variability in RSVF-multi-4 crystal structure 10
Supplementary Figure 9: RSVF-multi-4 crystal structure analysis of site-V graft 10
Supplementary Table 3: Crystallography collection and refinement statistics: 11
Supplementary Table 4: Sequences of experimentally characterized designs 12
Supplementary Table 5: Structural motif indices for RSVF site-II, IV, and V 13
Supplementary Table 6: Motif residues redesigned with ProteinMPNN for RSVF site-II, IV, and V 13

Supplementary Figures

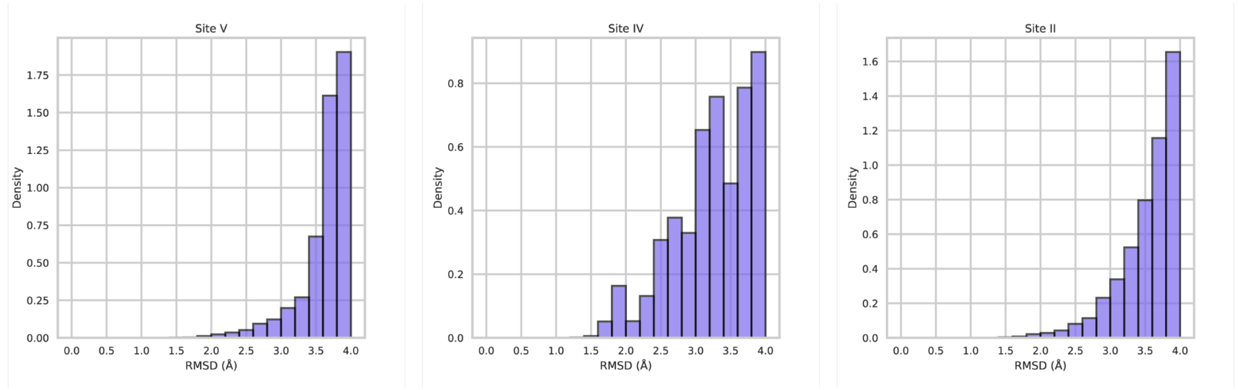

Supplementary Figure 1: Available natural scaffolds to host RSVF epitopes

A-C) Number of template structures in the PDB that can accommodate each motif targeted for scaffolding. A MASTER search was performed over a non-redundant PDB list containing a total of 8133 structures. The count of the structures recovered is plotted on the y-axis.

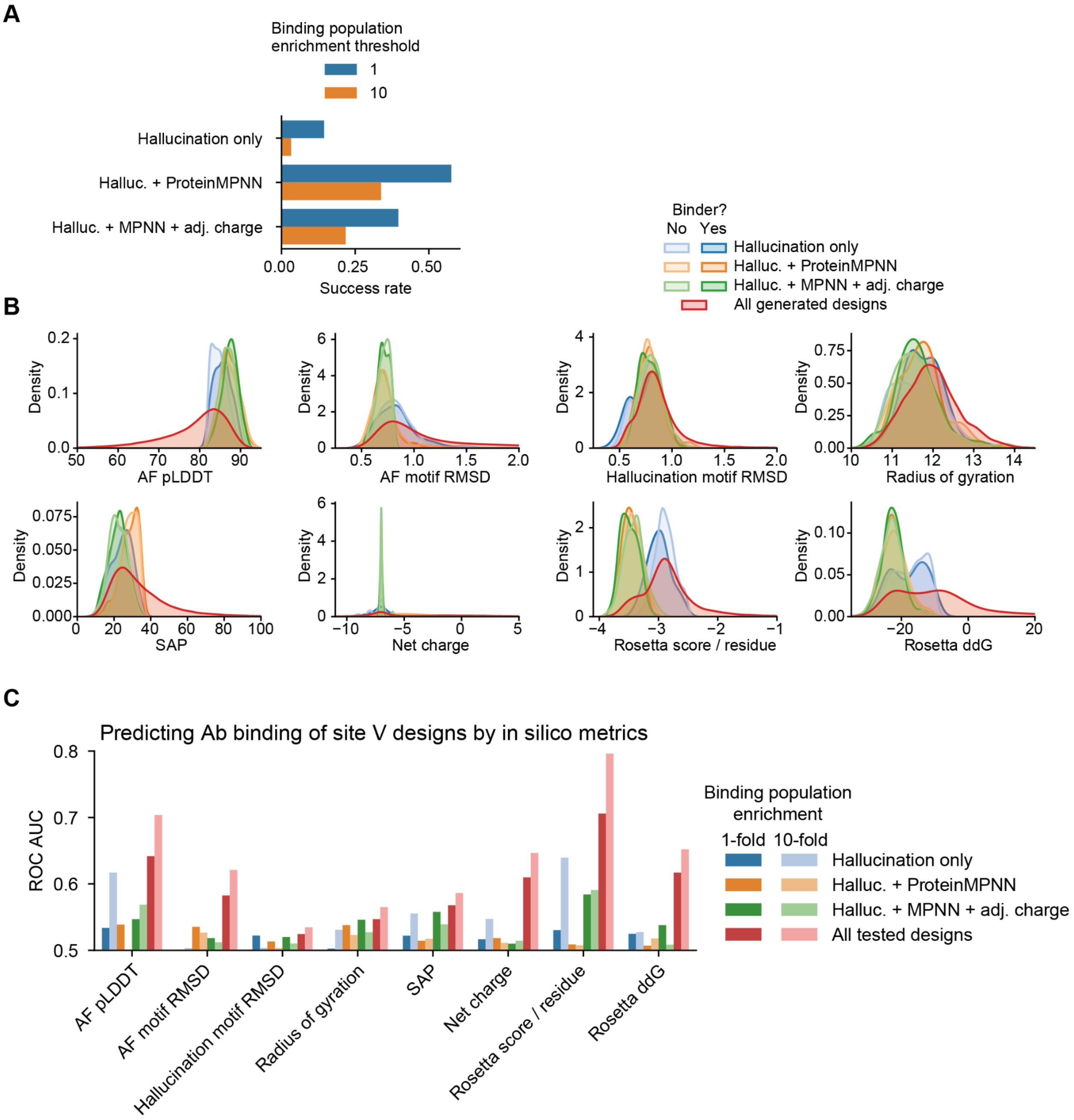

Supplementary Figure 2. Pooled analysis of RSVF site V designed binders

A) Success rates of RSVFV designs from the 3 design pipelines. **B)** Distributions of all *in silico* metrics used to filter designs prior to ordering for synthesis experimental testing. **C)** Receiver-operating-characteristic area under the curve for predicting binding success using each of the filtering metrics.

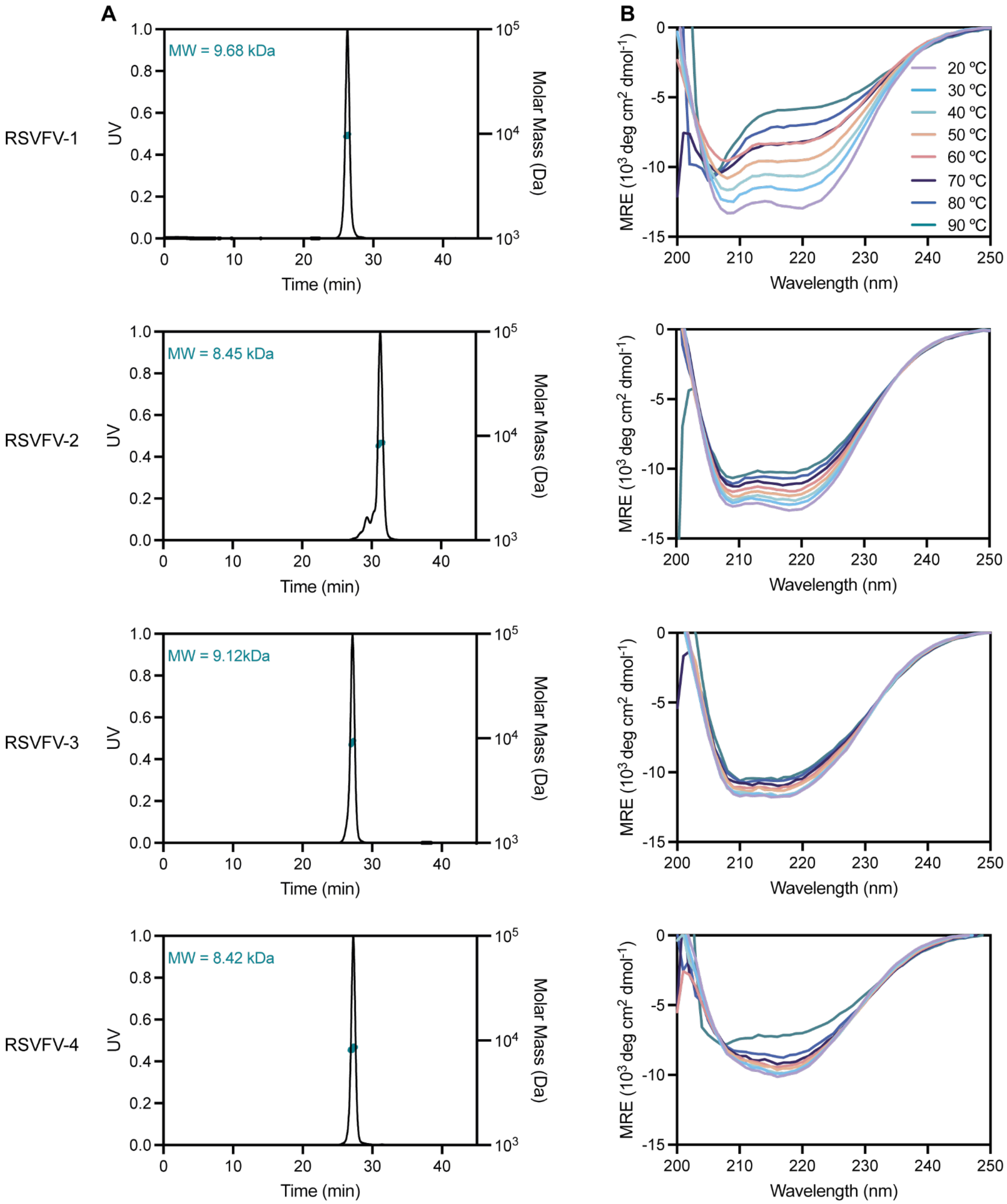

Supplementary Figure 3: RSVFV biochemical characterization

A) SEC-MALS measurement of oligomerization for each top candidate scaffold **C**) CD spectra at various incubation temperatures shown for each scaffold.

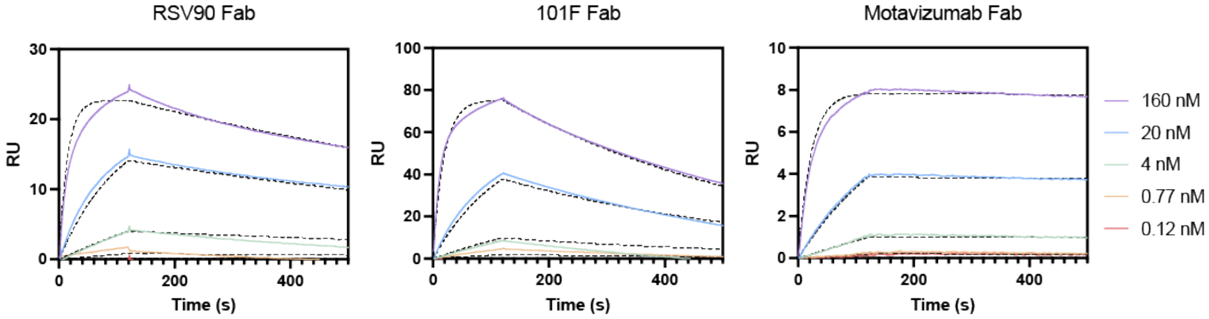

Supplementary Figure 4: RSVF trimer affinities

SPR affinity measurement of immobilized RSVF trimer against RSV90 Fab, 101F Fab, or Motazivumab Fab. Kinetics were fit using a 1:1 Langmuir model. Affinities to RSVF trimer: RSV90 Fab 0.9 nM, 101F Fab 2 nM, motavizumab Fab 15 pM

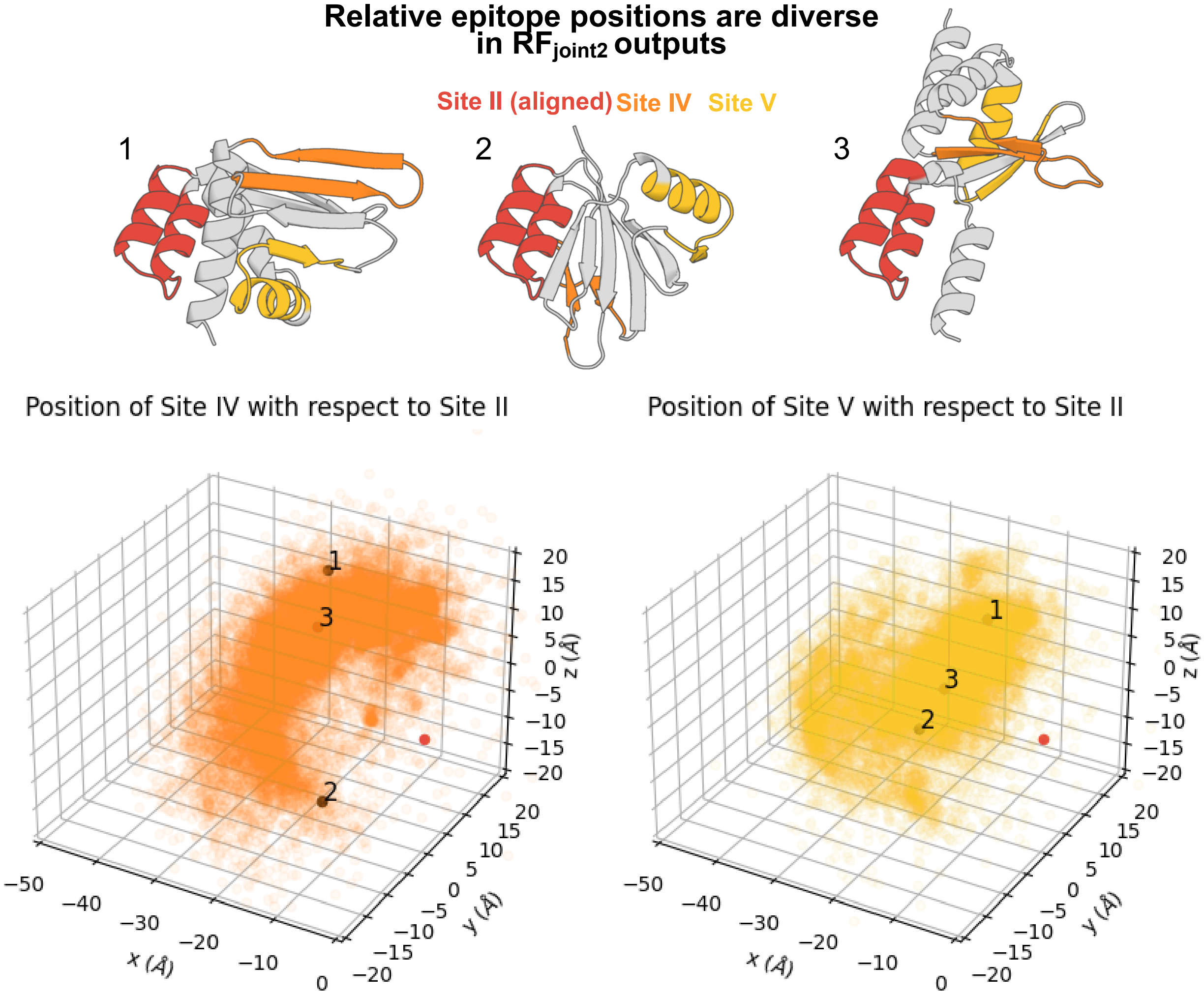

Supplementary Figure 5: RFjoint2 generates diverse inter-epitope positioning

A) As the relative position between epitopes is not fixed in RFjoint2, the multi-epitope designs have diverse inter-epitope positioning. Top row: three example *in silico* successful designs aligned to RSV-F Site II (red). Note the variation in the positioning of the Site IV and Site V epitopes (orange, yellow). Bottom row: quantification of the diversity of epitope positioning. Designs were aligned on RSV-F Site II, and the vector from the Site II center of mass (COM) to the COM of the other two epitopes was calculated. Red point indicates the COM of the aligned RSV-F Site II epitope in each design.

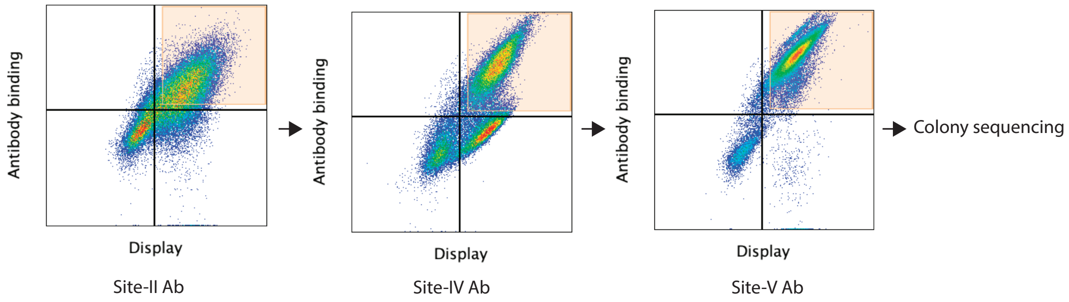

Supplementary Figure 6: Sequential sorting for the identification of multi-epitope scaffolds binding all target antibodies.

A) RSVF multi-epitope scaffold library screening by yeast display. Density plot of the library of scaffolds binding motavizumab antibody (site-II-specific) following proteolytic treatment. The binding population was sorted for subsequent screening against 101F antibody (site-IV-specific). The binding population was sorted for screening against RSV90 antibody (site-V-specific). The sorted population (orange square) is shown for each plot. Collected colonies binding all three antibodies were sequenced.

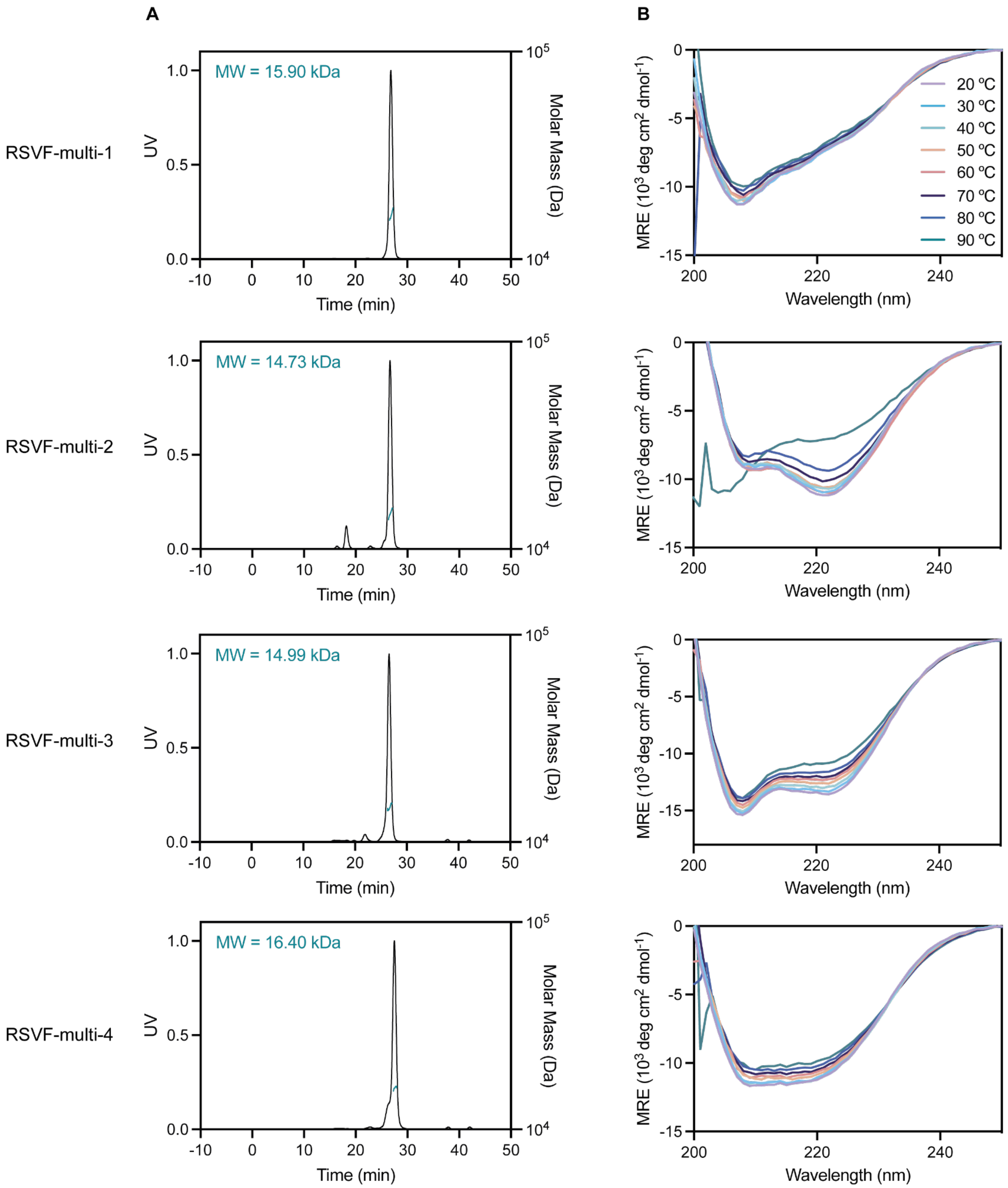

Supplementary Figure 7: RSVF-multi biochemical characterization

A) SEC-MALS measurement of oligomerization for each top candidate scaffold **C**) CD spectra at various incubation temperatures shown for each scaffold.

Supplementary Table 1: Affinity RSVFV and RSVF-multi designs site-specific antibodies

	**RSV90 Fab**	**101F IgG**	**Motavizumab IgG**	**RSV90 IgG**
**RSVFV-1**	54 nM ± 0.004			
**RSVFV-2**	108 nM ± 0.003			
**RSVFV-3**	94 nM ± 0.006			
**RSVFV-4**	241 nM ± 0.005			
**RSVF-multi-1**		522 nM ± 0.220	Not fitted	> 10uM
**RSVF-multi-2**		377 nM ± 0.293	47 nM ± 0.0009	> 10uM
**RSVF-multi-3**		343 nM ± 0.080	18 nM ± 0.006	> 10uM
**RSVF-multi-4**		890 nM ± 0.341	14 nM ± 0.003	> 10uM

SEM reported based on curve fitting

Supplementary Table 2: Structural accuracy of grafted RSVF epitopes on single and multi-motif scaffolds.

The RMSD of C_α, C, N, CO, and C_β atoms of grafted RSVF epitopes on scaffolds compared to crystal structures of RSVF native epitopes (PDB:5TPN).

Crystal Structure	RMSD (Å) Site-II	RMSD (Å) Site-V	RMSD (Å) Site-IV
RSVFV-1		0.843	
RSVF-multi-1	0.536	1.776	1.713
RSVF-multi-4	0.393	0.790	1.355

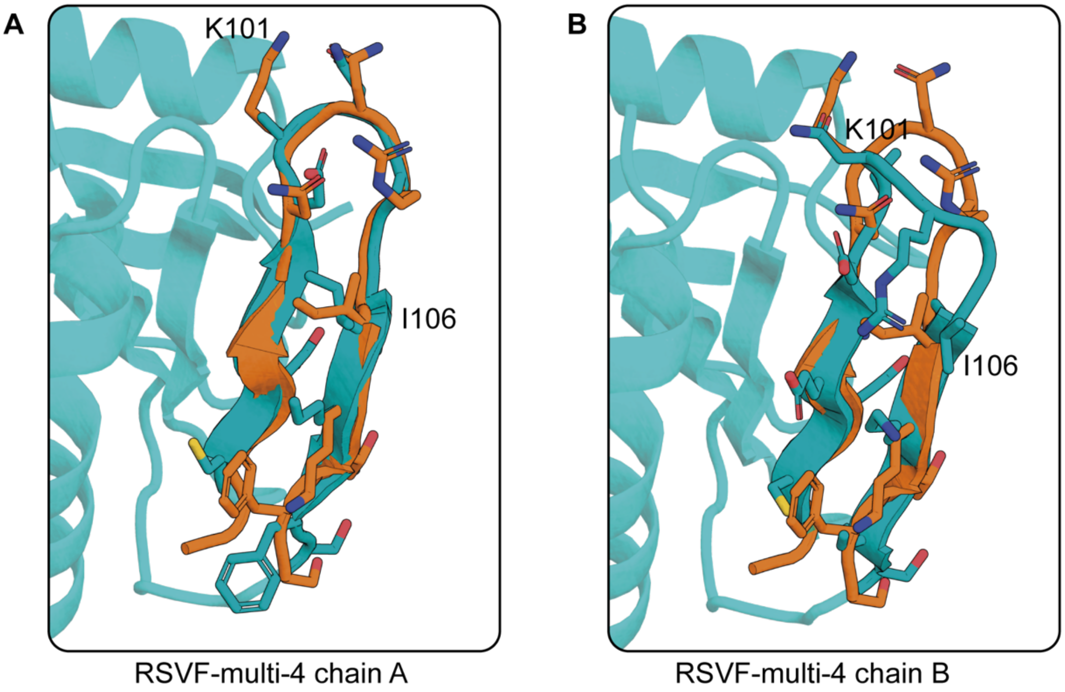

Supplementary Figure 8: Site-IV variability in RSVF-multi-4 crystal structure

Native site-IV (orange) (PDB: 3O45) overlaid on the crystal structure of RSVF-multi-4 (teal) A) Chain A residues 101-106 closely agree with the native epitope backbone. B) Chain B residues 101-106 deviate from the native epitope backbone.

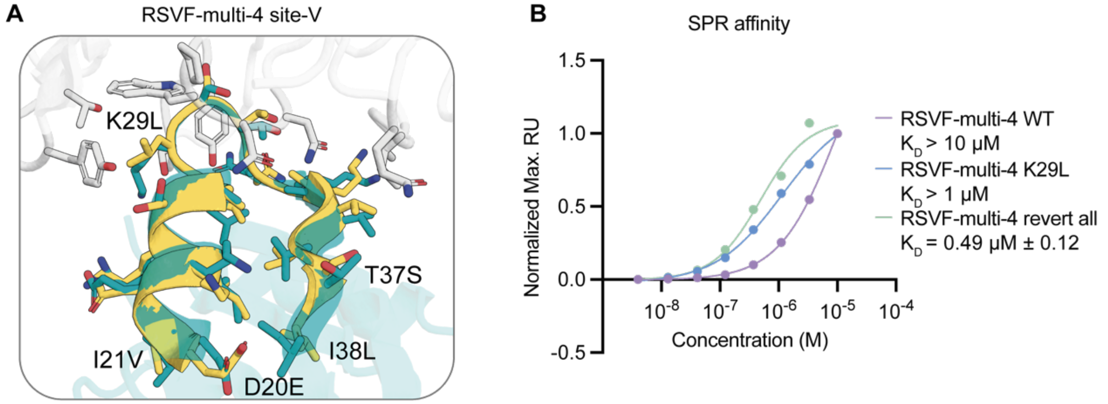

Supplementary Figure 9: RSVF-multi-4 crystal structure analysis of site-V graft

A) RSVF native site-V structure overlayed on RSVF-multi-4 site-V graft. Residues permitted to redesign during multi-epitope scaffolding are labelled with residue identity in RSVF-multi-4 and the corresponding native epitope residue. B) SPR steady state affinity measurements for the

RSVF-multi-4 scaffold (WT), the K29L mutant, and fully-reverted mutant against RSV90 IgG. SEM values reported based on curve fitting.

Supplementary Table 3: Crystallography collection and refinement statistics:

X-ray data collection and refinement statistics for RSVF-V and multi-epitope scaffold alone or in complex with their target antibodies.

	**RSVF-multi-4**	**Motavizumab fab/RSVF-multi-1**	**RSV90 Fab/RSVF-V H6**
Wavelength	0.96	0.87	1.00
Resolution range	77.67 - 2.3 (2.382 - 2.3)	78.23 - 2.91 (3.014 - 2.91)	73.83 - 2.43 (2.517 - 2.43)
Space group	P 21 21 21	C 1 2 1	P 21 21 2
Unit cell	46.843 106.432 113.589 90 90 90	184.072 66.859 109.41 90 103.865 90	113.39 137.65 87.47 90 90 90
Total reflections	135054 (13569)	86269 (8958)	332044 (34355)
Unique reflections	25945 (2558)	28467 (2829)	52204 (5153)
Multiplicity	5.2 (5.3)	3.0 (3.2)	6.4 (6.7)
Completeness (%)	99.26 (99.96)	99.67 (99.82)	99.79 (99.96)
Mean I/sigma(I)	9.17 (1.55)	6.14 (1.52)	9.58 (1.44)
Wilson B-factor	54.13	45.54	52.47
R-merge	0.08944 (0.9056)	0.1444 (0.7102)	0.1489 (2.189)
R-meas	0.09991 (1.005)	0.1753 (0.8547)	0.1623 (2.377)
R-pim	0.04371 (0.4287)	0.09815 (0.4709)	0.06398 (0.9186)
CC1/2	0.996 (0.77)	0.989 (0.738)	0.997 (0.58)
CC*	0.999 (0.933)	0.997 (0.921)	0.999 (0.857)
Reflections used in refinement	25819 (2558)	28586 (2828)	52183 (5152)
Reflections used for R-free	1357 (132)	1407 (142)	2632 (243)
R-work	0.2658 (0.3414)	0.2228 (0.3267)	0.2462 (0.4039)
R-free	0.2962 (0.3935)	0.2600 (0.3622)	0.2866 (0.4492)
CC(work)	0.940 (0.779)	0.941 (0.828)	0.946 (0.777)
CC(free)	0.923 (0.710)	0.910 (0.757)	0.946 (0.725)
Number of non-hydrogen atoms	3968	8365	7750

macromolecules	3908	8316	7704
ligands	0	4	0
solvent	60	45	46
Protein residues	493	1078	1016
RMS(bonds)	0.005	0.007	0.002
RMS(angles)	0.89	1.07	0.51
Ramachandran favored (%)	97.09	96.42	95.91
Ramachandran allowed (%)	2.91	3.58	3.89
Ramachandran outliers (%)	0	0	0.20
Rotamer outliers (%)	0.23	1.17	0.82
Clashscore	4.15	9.1	5.54
Average B-factor	66.14	50.71	74.65
macromolecules	66.28	50.71	74.75

Supplementary Table 4: Sequences of experimentally characterized designs

Name	Sequence	Expression vector
RSVFV-1	METEEEIIEKVKSALLSTNKAVISVELKGRTIPLYVEITKEGKLHLTAEGATEEEKEIIKEAQKAFQEEIEHEAERKEK	pet11b
RSVFV-2	SAELDVKAEAIVNKIESALLSTNKAVVSWEGKTLTVTLENNTLIIEVEEVDEEMKELLEKAAKLWEEKKGKKAEVILP	pet11b
RSVFV-3	MVTKEEIINKIKSALLSTNKAVVSIKNPKTNEYVPFLVTNNGGEIVVEDTNGNKFVSKNSLEDVANWILEYLK	pet11b
RSVFV-4	MTPEEAKELYEKAKSALLSTNKAVISAEINGKTLTAEVSLTSDNKIEVTITEGDKTTTITFDTNDENKYTTEETSA	pet11b
RSVF-multi-1	MKLVIARVKSPKVKRLSEEDIEKIKSALKSTNKAVVTIKDENGEEIEVEVRLLTLEEALKYINDLPISNDAKKLMSNNIHKALEPGRTVVFGPEGCEERDKNRGIIKTFSTDVKLDETYFFFRVE	pet11b
RSVF-multi-2	MKLVIARVKSPKVKRLSEEDIEKIKSALKSTNKAVVTIKDENGEEIEVEVRLLTLEEALKYINDLPISNDAKKLMSNNIHKALEPGRTVVFGPEGCEERDKNRGIIKTFSTDVKLDETYFFFRVE	pet11b

RSVF-multi-3	DDLVDIFLRAFAKAAKVTRFDKNRGIIKTFSEEETEKLFKSLTEEEV EKIESALKSTNKAVVVLGDGDIEIEIDLDKLYALINDLDISNDQKKEM SNNFFEYLRKIAKK	pet11b
RSVF-multi-4	MKLVIARVKSPKVKRLSEEDIEKIKSALKSTNKAVVTIKDENGEEIEV EVRLLTLEEALKYINDLPISNDAKKLMSNNIHKALEPGRTVVFGPEG CEERDKNRGIIKTFSTDVKLDETYFFFRVE	pet11b

Supplementary Table 5: Structural motif indices for RSVF site-II, IV, and V

**Site**	**Residues**
II	F255-277
IV	F420-436
V	F163-181

Supplementary Table 6: Motif residues redesigned with ProteinMPNN for RSVF site-II, IV, and V

**Site**	**Residues permitted for redesign**
II	F255-261,F264-267,F270,F273-274,F277
IV	None
V	F163-168,F171-172, F177, F179-181
