# Supplementary material for: Accurate single-domain scaffolding of three nonoverlapping protein epitopes using deep learning
Source: Nat Chem Biol. 2025 Dec 5;22(4):604–11. doi: 10.1038/s41589-025-02083-z (PMC13038408; doi:10.1038/s41589-025-02083-z)
Supplement: Supplementary file 2 — Reporting Summary [file 41589_2025_2083_MOESM2_ESM.pdf]

nature portfolio

Corresponding author(s): Bruno Emanuel Correia

Last updated by author(s): 20/10/25

Reporting Summary

Nature Portfolio wishes to improve the reproducibility of the work that we publish. This form provides structure for consistency and transparency in reporting. For further information on Nature Portfolio policies, see our Editorial Policies and the Editorial Policy Checklist.

Statistics

For all statistical analyses, confirm that the following items are present in the figure legend, table legend, main text, or Methods section.

n/a	Confirmed	
☐	☒	The exact sample size (*n*) for each experimental group/condition, given as a discrete number and unit of measurement
☐	☒	A statement on whether measurements were taken from distinct samples or whether the same sample was measured repeatedly
☒	☐	The statistical test(s) used AND whether they are one- or two-sided<br>*Only common tests should be described solely by name; describe more complex techniques in the Methods section.*
☒	☐	A description of all covariates tested
☒	☐	A description of any assumptions or corrections, such as tests of normality and adjustment for multiple comparisons
☐	☒	A full description of the statistical parameters including central tendency (e.g. means) or other basic estimates (e.g. regression coefficient) AND variation (e.g. standard deviation) or associated estimates of uncertainty (e.g. confidence intervals)
☒	☐	For null hypothesis testing, the test statistic (e.g. *F*, *t*, *r*) with confidence intervals, effect sizes, degrees of freedom and *P* value noted<br>*Give P values as exact values whenever suitable.*
☒	☐	For Bayesian analysis, information on the choice of priors and Markov chain Monte Carlo settings
☒	☐	For hierarchical and complex designs, identification of the appropriate level for tests and full reporting of outcomes
☒	☐	Estimates of effect sizes (e.g. Cohen's *d*, Pearson's *r*), indicating how they were calculated

Our web collection on statistics for biologists contains articles on many of the points above.

Software and code

Policy information about availability of computer code

Data collection	Research code has been made available at https://github.com/RosettaCommons/RFjoint2/
Data analysis	Flow cytometry analysis: Sony SH800 (2.1.5), FloJo(10.8.1))<br>Biochemistry: GraphPad Prism(10.2.3), Biacore 8k evaluation software(4.0.8.19879) for SPR data, Chromeleon(7.2.10) for MALS data<br>Crystal structures: Coot(0.9.8.7), Phenix (1.20.1-4487-00), PyMol (2.0)

For manuscripts utilizing custom algorithms or software that are central to the research but not yet described in published literature, software must be made available to editors and reviewers. We strongly encourage code deposition in a community repository (e.g. GitHub). See the Nature Portfolio guidelines for submitting code & software for further information.

Data

Policy information about availability of data

All manuscripts must include a data availability statement. This statement should provide the following information, where applicable:

- Accession codes, unique identifiers, or web links for publicly available datasets
- A description of any restrictions on data availability
- For clinical datasets or third party data, please ensure that the statement adheres to our policy

Structures have been deposited in the Protein Data Bank under accession codes 9F91 (RSVFV-1 in complex with RSV90 Fab), 9F90 (RSVF-multi-1 in complex with motavizumab Fab), and 9F8Y (RSVF-multi-4). Protein sequences are available in Supplementary Table 4. The plasmids of the designed proteins are available from the authors under a material transfer agreement with the Ecole Polytechnique Fédérale de Lausanne (EPFL). All code used for this study will be made available.

Human research participants

Policy information about studies involving human research participants and Sex and Gender in Research.

Reporting on sex and gender	*Use the terms sex (biological attribute) and gender (shaped by social and cultural circumstances) carefully in order to avoid confusing both terms. Indicate if findings apply to only one sex or gender; describe whether sex and gender were considered in study design whether sex and/or gender was determined based on self-reporting or assigned and methods used. Provide in the source data disaggregated sex and gender data where this information has been collected, and consent has been obtained for sharing of individual-level data; provide overall numbers in this Reporting Summary. Please state if this information has not been collected. Report sex- and gender-based analyses where performed, justify reasons for lack of sex- and gender-based analysis.*
Population characteristics	*Describe the covariate-relevant population characteristics of the human research participants (e.g. age, genotypic information, past and current diagnosis and treatment categories). If you filled out the behavioural & social sciences study design questions and have nothing to add here, write "See above."*
Recruitment	*Describe how participants were recruited. Outline any potential self-selection bias or other biases that may be present and how these are likely to impact results.*
Ethics oversight	*Identify the organization(s) that approved the study protocol.*

Note that full information on the approval of the study protocol must also be provided in the manuscript.

Field-specific reporting

Please select the one below that is the best fit for your research. If you are not sure, read the appropriate sections before making your selection.

☒ Life sciences ☐ Behavioural & social sciences ☐ Ecological, evolutionary & environmental sciences

For a reference copy of the document with all sections, see nature.com/documents/nr-reporting-summary-flat.pdf

Life sciences study design

All studies must disclose on these points even when the disclosure is negative.

Sample size	4,547 RSVF-V designs and 32 RSV-multi designs were screened by yeast display.
Data exclusions	For next generation sequencing only high quality reads containing the correct protein translation frame were used for analysis. Neutralization RSVF replicate 1 cell assay column 8 did not lyse as confirmed by virus only control, thus removed from processing
Replication	Biophysical characterization measurements from independent protein preparations were consistent. Cellular assay was performed three times for both samples plotted
Randomization	Randomization was not performed for this study
Blinding	Blinding was not relevant to this study. Computational designs passed through extensive screening and biophysical characterization

Reporting for specific materials, systems and methods

We require information from authors about some types of materials, experimental systems and methods used in many studies. Here, indicate whether each material, system or method listed is relevant to your study. If you are not sure if a list item applies to your research, read the appropriate section before selecting a response.

Materials & experimental systems

n/a	Involved in the study
☐	☒ Antibodies
☐	☒ Eukaryotic cell lines
☒	☐ Palaeontology and archaeology
☐	☒ Animals and other organisms
☒	☐ Clinical data
☒	☐ Dual use research of concern

Methods

n/a	Involved in the study
☒	☐ ChIP-seq
☐	☒ Flow cytometry
☒	☐ MRI-based neuroimaging

Antibodies

Antibodies used	RSVF monoclonal antibodies were produced in-house by transient transfection in HEK293 cells<br>Antibodies for flow cytometry were used at 1:100 dilution<br>PE-conjugated anti-Fab (Thermo Scientific, #MA1-10377)<br>PE-conjugated anti-human Fc (BioLegend, #342303)<br>FITC-conjugated anti-HA (Bethyl, A190-138F)
Validation	Antibodies were produced in-house using protein A affinity resin and further purification by size exclusion chromatography. Binding to RSVF pre-fusion protein was confirmed by SPR

Eukaryotic cell lines

Policy information about cell lines and Sex and Gender in Research

Cell line source(s)	HEK293 expression cells Thermo Fisher Scientific (A14635)<br>ExpiCHO-S Cells Thermo Fisher Scientific (#A29127)<br>HEP2 cells were a gift of Marie-Anne Rameix-Welti, UFR des Sciences et de la Santé, Paris
Authentication	Cell lines were not further authenticated
Mycoplasma contamination	Cells were not tested for mycoplasma contamination
Commonly misidentified lines<br>(See ICLAC register)	No commonly misidentified lines were used in this study

Animals and other research organisms

Policy information about studies involving animals; ARRIVE guidelines recommended for reporting animal research, and Sex and Gender in Research

Laboratory animals	Female BALB/C mice at 5-weeks old were obtained from Janvier labs and acclimatized for one week.
Wild animals	The study did not involve wild animals
Reporting on sex	Sex based analysis were not performed. All mice used in this study were female for caging purposes
Field-collected samples	The study did not involve field-collected samples
Ethics oversight	All animal experiments were approved by the Vaud Veterinary Cantonal Authorities in accordance with Swiss regulations of animal welfare (VD3808).

Note that full information on the approval of the study protocol must also be provided in the manuscript.

Flow Cytometry

Plots

Confirm that:

☒ The axis labels state the marker and fluorochrome used (e.g. CD4-FITC).

☒ The axis scales are clearly visible. Include numbers along axes only for bottom left plot of group (a 'group' is an analysis of identical markers).

☒ All plots are contour plots with outliers or pseudocolor plots.

☒ A numerical value for number of cells or percentage (with statistics) is provided.

Methodology

Sample preparation	Cells were induced overnight. Pellets corresponding to 2 mL at OD600 of 1 were washed once and resuspended 250 uL in TBS (20 mM Tris pH 8.0, 150 mM NaCal). Final concentrations of chymotrypsin were 0.01 uM (pCTcon2 vector) or 0.1 uM trypsin (pNTA-V5 vector). Chymotrypsin was added to the resuspended yeast and incubated at room temperature for 5 minutes. The reaction was quenched in cold PBS + 2 % BSA. Cells were pelleted and washed in cold wash buffer (PBS + 0.05% BSA) three times. Cells were labelled with 1 µM of the target (RSV90 Fab for RSVFV library, motavizumab IgG for the RSVF-multi library) at 4 °C for 2 hours. Cells were washed twice with wash buffer and then incubated with FITC-conjugated anti-HA and PE-conjugated anti-human Fc or PE-conjugated anti-Fab for an additional 30 minutes. Subsequent sorts were not subject to limited proteolysis.
Instrument	Sony SH800
Software	Collection Sony SH800<br>Analysis FloJo
Cell population abundance	Cells were sorted in ultra-purity mode. After three subsequent sorts with three different antibodies, cells were plated and single clones were sequenced
Gating strategy	FSC/SSC gating was applied to only analyze single cells. Double positive populations FITC for surface display and PE for antibody binding were sorted as shown in supplementary figure 6

☒ Tick this box to confirm that a figure exemplifying the gating strategy is provided in the Supplementary Information.
